# Whose School Is Safe? Informant-Specific Validity in School Safety and Student Well-Being Assessment Across 63 Education Systems

**DOI:** 10.3390/healthcare14172848

**Published:** 2026-09-04

**Authors:** Georgios Sideridis, Mohammed Alghamdi

**Affiliations:** 1 Boston Children’s Hospital, BARD, Harvard Medical School, 300 Longwood Avenue, Boston, MA 02115, USA; 2 Department of Self-Development Skills, King Saud University, Riyadh 11451, Saudi Arabia; mhalghamdi@ksu.edu.sa

**Keywords:** school safety, school health, student well-being, peer victimization, school belonging, informant discrepancy, multitrait–multimethod, response surface analysis, TIMSS

## Abstract

**Highlights:**

**What are the main findings?**

- Child, teacher, and principal reports of school safety obtained in the same schools were close to statistically independent; neither ceiling effects in the adult reports nor unreliability of the school-level aggregates accounted for the weak convergence.
- Informant validity was domain-specific. Child reports accounted for the majority of the informant-explained variance in school belonging and in peer victimization, but only for approximately one third in mathematics and science achievement, where the three informants contributed comparably. The three reports jointly explained between 1% and 13% of between-school variance, so these proportions were computed on a modest total.

**What are the implications of the main findings?**

- Adult reports of institutional conditions cannot substitute for children’s reports of experienced safety; school-health surveillance should therefore include student self-reports.
- Child, teacher, and principal indicators should be reported separately. Aggregates from approximately 30 students can support school-level monitoring with uncertainty intervals but should not yet be treated as validated screening measures.

**Abstract:**

Background/Objectives: Schools constitute the principal setting for the identification of children’s psychosocial risk and the delivery of preventive support, and school-health surveillance therefore depends on informant reports. Selection of the reporting informant is constrained by feasibility, cost, and questionnaire length, which frequently preclude multi-informant assessments, and the question of whether adult and child reports index a common property has predominantly been examined in single-country designs. The present study estimated the criterion-related validity of child, teacher, and principal reports of school safety across multiple education systems, against outcomes spanning children’s experience, institutional functioning, and externally assessed achievements; the estimand was thus informant-specific validity rather than cross-informant agreement. Methods: TIMSS 2023 Grade 4 data from 12,955 schools in 63 education systems (395,961 children, 27,100 teacher records) were analyzed, with child, teacher, and principal reports modeled as separate informants. Students were randomly split within schools so that no student contributed both a predictor and an outcome; the split was replicated 50 times and averaged. Convergence was evaluated with a multitrait–multi-informant model, with ceiling effects and differential unreliability specified as rival explanations; informant contributions were estimated by an exact relative-importance (LMG) variance decomposition, with response surface analysis reported as a secondary characterization of directional discrepancy. Results: Median school-level cross-informant correlations were below 0.10 and were not attributable to ceiling effects or to unreliability. With all three informants retained, the child report accounted for 95% (95% CI 92–97) of the informant-explained variance in school belonging and 75% (65–82) in peer victimization, but 44% (28–59) in mathematics, where the three contributions were approximately equal. These proportions were computed on a modest total: the three informant scores jointly explained between 1% and 13% of between-school variance. The pattern was robust to informant, item, covariate, and weighting specifications, and to correction for differential predictor unreliability. Conclusions: Criterion-related informant validity in school safety measurement was domain-specific. For outcomes describing children’s experience, adult reports contributed substantially lesser than child reports and cannot be regarded as interchangeable substitutes for children’s reports of experienced safety. Because school-health surveillance, risk identification, preventive targeting, and resource allocation depend on these inputs, valid monitoring requires student self-reports rather than adult proxy reports alone.

## 1. Introduction

Every school-health and safety monitoring system rests on a measurement assumption that is seldom examined. Whether the instrument is a state accountability indicator, a school inspectorate rubric, a health-promoting-school audit, or an international assessment questionnaire, the operative question—whether a given school is safe—is resolved by obtaining a report from a designated informant. Informant selection has been governed less by measurement considerations than by feasibility. Principals are surveyed because one is available per school; teachers because they are already within the sampling frame; and children least frequently, because student administration requires instructional time, consent procedures, and age-appropriate instrumentation, and because student items are the first to be removed when questionnaire length is constrained. These constraints are substantive: time, cost, and respondent burden materially limit the number of perspectives a surveillance system can accommodate, and single-informant practice is in part a reasonable response to them. A selection that is defensible on feasibility grounds nevertheless carries an untested measurement assumption, and it is that assumption, rather than the constraint, that the present study examined.

That ordering would be defensible if the informants were substitutable. The present study tested that assumption in 63 education systems and did not support it, although the pattern of failure differed from the one commonly assumed. Adult reports were not globally uninformative: they carried substantially more information about some outcomes than about others, and the outcome domain at which their informativeness changed had direct implications for the monitoring of children’s well-being.

### 1.1. School Safety as a Health and Well-Being Problem

School safety is a health concern as much as an educational one. Most lifetime mental disorders emerge during the school years—about half by age 14 and roughly a third of young people worldwide meet the criteria for a disorder before adulthood [1,2]—and schools are, in practice, the setting in which children’s psychosocial difficulties are most often identified and in which mental-health services are most often delivered [3]. Health-promoting-school frameworks accordingly treat the psychosocial environment as a determinant of child health alongside nutrition, physical activity, and health services, and whole-school programs built on them can reduce risk behavior and improve well-being [4]—but only where that environment can be assessed with sufficient accuracy to support intervention. The associated risks are well documented. Peer victimization is associated with concurrent and later academic difficulty [5] and, across adolescence, with depression, anxiety, self-harm, and suicidal ideation [6], with reduced school belonging [7,8], and with psychosocial harms that anti-bullying programs have shown limited efficacy in reducing [9,10]. Conversely, school connectedness is among the most robust modifiable protective factors for adolescent and even adult health, predicting lower emotional distress, violence, and substance use years later [11,12]; and school climate is implicated in violence and attainment in longitudinal designs [13] and moderates the association between disadvantage and achievement [14].

Surveillance systems built on these frameworks must make an informant choice, and that choice determines what can be detected. Three features of school safety make the choice consequential. First, the construct is experiential: a perceived lack of safety is a state of the individual student rather than a property of the physical or institutional setting. Much peer victimization occurs in forms that are not observable to supervising adults [15], and the forms hardest for adults to see—exclusion, rumor spreading, and electronically mediated aggression—are precisely those most resistant to intervention. Second, the incentives are asymmetric: reported safety is an accountability variable with consequences for inspection ratings and leadership tenure, so a principal reporting on their own school occupies a structurally different reporting position from a fourth-grade student reporting on their own recent experience. No intentional misreporting need be assumed. Third, the resulting measurement failure is undetectable within the system that produces it: a school in which adults do not perceive a safety problem and students do is precisely the school that adult-based monitoring will not identify.

The choice of informant therefore propagates into four downstream functions of school-health systems that depend on these data: the population surveillance of student well-being, identification of schools and children at psychosocial risk, targeting of preventive interventions, and allocation of scarce health and counseling resources [12,16,17]. The practical problem is thus specific: if school-health systems rely on adult reports, they may classify institutional order accurately while failing to identify schools in which children experience victimization, disconnection, or fear. Whether that risk is realized is an empirical question about informant validity, and it is the question that the present study addresses.

### 1.2. Informant Discrepancies as Substantive Rather than Erroneous

The assumption of informant substitutability was abandoned in developmental psychopathology four decades ago. Achenbach et al. [18] found a mean cross-informant correlation of 0.28 between different types of informants against 0.60 between informants of the same type, and concluded that reports were situation specific. Subsequent work established that discrepancies were systematic and informative: informants observed children in different contexts, applied different comparison standards, and occupied different positions relative to what was reported [19,20,21]. The Operations Triad Model [22] formalizes the consequence: a discrepancy may reflect measurement errors or genuinely context-dependent behaviors, and the two have opposite implications for practice. Where the interpretation is substantive, the appropriate response is not to average informants but to model their reports jointly [23].

An implication that has been underexploited is that if discrepancies are contextual, informant validity should be domain-specific: each informant should be most valid for outcomes falling within the context they observe. This is a stronger and more falsifiable claim than the proposition that informants disagree, and it is the claim that the present design tested.

School climate research has repeatedly encountered informant divergence without pursuing this implication. Mitchell et al. [24] documented a systematic divergence between student and teacher perceptions in the same schools; Konold et al. [25] and Bradshaw et al. [26] developed multi-informant climate instruments in which student and adult reports formed related but distinguishable factors; and Cornell and Huang [27] showed that student-perceived climates predicted risk behavior. Yet the two major reviews of the construct [28,29], and a recent review of school-climate assessments [30], give little attention to whose climate is being measured, and the modal response in applied work is to treat divergence as unreliable, to average across informants, or to select a single informant.

Student self-reports are extensively studied and well validated for a wide range of school and psychosocial outcomes [25,27,30]. The gap is comparative and criterion-related, and it has two parts. First, informant reports have rarely been evaluated against one another on criterion-related terms—by entering the informants simultaneously and asking whose report predicts which outcome, with the outcome domain varied systematically—rather than on convergent terms, by asking how strongly the reports correlate with each other. Convergence and validity are distinct questions and only the latter is informative for informant selection in surveillance. Second, the multi-informant school-climate evidence base is built almost entirely within single countries, so it cannot establish whether the relative standing of child and adult reports is a property of the measurement or of a particular national context, nor quantify how far that standing varies across systems. The present study addressed this gap by testing domain-specific informant validity across many education systems simultaneously.

### 1.3. Content and Referents of the Three Informant Questionnaires

Any multi-informant comparison of this kind confronts a problem that must be stated at the outset because it constrains what the results can mean. The TIMSS questionnaires were not written for a multi-informant study, and the items carrying the word safety do not share a referent. Children are asked whether they feel safe at school, whether their teachers care about them, and whether students like each other. Teachers are asked whether they feel safe and whether the school has effective security policies. Principals are asked how much of a problem intimidation, physical fights and the intimidation of teachers are. A child rating personal safety and relatedness, a teacher rating their own safety and a policy, and a principal rating the incidence of disciplinary events are not parallel measurements of one construct, and the low convergence among them reflects some unknown combination of informant perspective, referent, item content, level of observation, and measurement error. The principal consequence is stated explicitly at the outset. Because the adult items index the school as an institution while the child items index the child’s own experience of being in it, informant and construct coverage are confounded by the instrument: any result reported below is jointly informative about informant identity and about the content of the items available for that informant. Section 4.1 distinguishes the two resulting interpretations and specifies the inferences each supports.

Three design features mitigate this limitation without resolving it. First, the comparison that carries the discriminant argument is internal to the multitrait–multi-informant matrix: it asks whether one informant’s ratings of two acknowledged different constructs cohere more strongly than two informants’ ratings of the nominally same construct. Second, every substantive model is re-estimated using the single child item whose referent most closely matches the teacher item—I feel safe at school—which is also content-independent of the belonging outcome. Third, and most importantly, the outcome was varied rather than only the predictor, so that the question addressed was not whether informants agreed but what each informant’s report was valid for. Referent heterogeneity cannot account for the differential predictive validity of a single informant’s report across outcomes.

### 1.4. Four Threats to a Multi-Informant Comparison

The validity of a multi-informant comparison depends on its protection against four artifacts. Each can generate the appearance of an informant difference where none exists, or mask one that does, and each therefore forms part of the inferential argument rather than an ancillary caveat. Each is addressed below by a specific design feature.

The first two threaten the size of any cross-informant association. A school-level score built from respondents is a group mean, and a group mean is unreliable when the intraclass correlation is small and the number of respondents is modest [31,32,33]; it is ICC(2), not ICC(1), that governs the resulting attenuation [34,35]. Critically, this unreliability is differential rather than uniform—severe for teachers, whose school aggregate rests on roughly two raters; moderate for children; and of an entirely different kind for principals, for whom no rater-sampling component exists. Differential attenuation can distort not only how large the cross-informant correlations are but which of them is largest, and the comparison among them is the quantity on which the present analysis depends. Range restriction acts in the same direction: if adults cluster at the top of a short scale, as roughly half of them were in the present data, any correlation involving them is attenuated by truncation rather than by genuine disagreement. Neither artifact was dismissed; both were specified as rival explanations and tested against the substantive account.

The remaining two threaten the interpretation of an association once its magnitude is established. Regressing a student-reported outcome on a student-reported predictor aggregated from the same children confounds a school effect with a method effect, because the same raters contribute to both the predictor and the outcome [36,37]; this confound is addressed by design, through random splitting of the student sample within schools, rather than by argument. And because the estimand is cross-national, each informant’s scale must carry a comparable meaning across dozens of systems before any cross-system statement is interpretable. Exact invariance across that many groups is implausible and conventional criteria behave poorly as the number of groups grows [38,39,40]; alignment optimization was developed for precisely this situation [41,42,43].

Taken together, these four considerations determined the design rather than merely qualifying it: teachers and principals were retained as separate informants rather than combined into an adult composite, the student sample was split within schools, reliability was reported as separable components rather than as a single coefficient, and comparability was established within informants before any cross-system comparison was undertaken. The research questions stated in Section 1.5 follow this order.

### 1.5. The Present Study

Three strands of the preceding literature converge on a single testable proposition. The developmental literature establishes that informant discrepancies are substantive rather than erroneous, and that their substance lies in the context each informant occupies [18,19,20,21,22]. The school-climate literature establishes that student and adult reports of the same school are related but distinguishable, without developing the implications for validity [24,25,26,27,28,29,30]. The measurement literature establishes that any answer must survive aggregation unreliability, ceiling effects, shared-rater variance and cross-national noncomparability [31,32,33,34,35,36,37,38,39,40,41,42,43]. Taken jointly, these establish that informant validity is not a general property of an informant but a joint property of the informant and outcome: each informant’s report should be most valid for outcomes falling inside the context that informant observes, and least valid for those falling outside it. This proposition, rather than the weaker claim that informants disagree, was the hypothesis the present design tested. It is falsifiable in that it predicts that the relative standing of child and adult reports should converge, or reverse, as the outcome moves outside the domain of children’s direct experiences.

The study addressed three questions, the second in two parts, and reported one further prespecified check. Each question corresponds to a single inferential decision, and the results are presented in the same order.

RQ1 (Comparability). Is each informant’s scale approximately invariant across the 63 education systems so that cross-system comparison is interpretable?

RQ2a (Convergence). Do child, teacher and principal reports of school safety converge at the school level and does any convergence between two informants exceed the correlation between two distinct constructs rated by the same informant?

RQ2b (Rival explanations). If convergence is weak, is that weakness attributable to ceiling effects in the adult reports or to differential unreliability of the school-level aggregates?

RQ3 (Domain-specific validity). Does the share of criterion variance attributable to each informant depend on the outcome predicted? Specifically, does the adult share increase as the outcome moves from children’s experiences (belonging, peer victimization) through institutional functioning (classroom disorder, absenteeism) to externally assessed achievements (mathematics and science)?

Teachers and principals were treated as separate informants throughout the primary analyses; a design whose central claim concerns the absence of a general adult perspective cannot coherently average adult reports. An adult composite was reported only as a secondary summary. Although the present data contain no clinical endpoints—victimization and belonging are treated throughout as psychosocial well-being indicators rather than as mental-health outcomes—the question is fundamentally one of health measurement, since it concerns which informant’s report constitutes a valid input to the surveillance, screening, prevention, and resource-allocation decisions required of school-health systems.

## 2. Materials and Methods

### 2.1. Sample and Design

The TIMSS 2023 Grade 4 international database [44] was analyzed; it was designed and administered according to the published assessment frameworks [45] and technical procedures [46]. All 63 participating countries and benchmarking entities with student, teacher and school questionnaire files were included. The analytic sample comprised 12,955 schools and 395,961 students. The teacher file contained 27,100 records; because TIMSS stored one record per teacher–class link, a teacher of two sampled classes appeared twice. Deduplication on country, school, and teacher identifier yielded 24,887 unique teachers (8.2% of records were duplicates), and all teacher aggregates were computed on unique teachers. These were sampled corresponding to the teachers associated with the sampled classes rather than a probability sample of all teachers in the school. The mean number of unique responding teachers per school was 1.92. In total, 11,371 schools (87.8%) had all three informants (Table 1). TIMSS uses a two-stage stratified cluster design in which schools are sampled with a probability proportional to size and intact classes are sampled within schools. The school was the unit of analysis. A complete listing of the 63 education systems, with school, student, unique-teacher, and principal counts and informant availability, is provided in Table S1.

### 2.2. Measures

**Safety.** Children: I feel safe at school, my teachers care about me, students at my school like each other (ASBG13B, D, G). Teachers: I feel safe at this school, this school has effective security policies (ATBG07A, B). Principals: Degree of problem of intimidation among students, physical fights, intimidation of teachers (ACBG14H, I, J). Because the three-item child scale combined personal safety with relatedness, every model was re-estimated with ASBG13B alone as the referent-matched child predictor. Two teacher items served as the substantive predictors, these being the two whose referent was safety. The alignment analysis (Table S3) used the full four-item safety-and-security block from the teacher questionnaire instead—ATBG07A and B plus This school has clear rules about student conduct (F) and The rules are enforced (G)—because alignment required a block of sufficient length to estimate loadings and intercepts. The differing item counts in Table 2 and Table S3 reflect this distinction.

**Order.** Children: How often the teacher must keep telling students to follow rules and how often others’ behaviors disrupt the lesson, in mathematics and science (ASBM04E, F; ASBS09E, F). Teachers: ATBG07C–E. Principals: ACBG14A–C.

**Outcomes.** Four child-reported outcomes spanned personal experiences and institutional functioning: peer victimization (11 items, ASBG14A–K), school belonging (ASBG13A, C, E, F), classroom disorder (the reverse of the child order items), and absenteeism (ASBG08). Two externally assessed outcomes were mathematics and science achievement. Achievement was not averaged across plausible values, because averaging discarded the imputation variance that plausible values were constructed to represent. Each of the five mathematics and five science plausible values was aggregated to the school separately and analyzed in turn, and the results were combined by Rubin’s rules. Holding the outcome informant constant across the first four outcomes rendered the domain comparison interpretable. Victimization and belonging were treated as psychosocial well-being indicators rather than as mental-health outcomes; TIMSS contained no health measures.

**Covariates.** All models controlled for the percentage of girls. Extended specifications added school-mean books in the home, home possessions, the frequency of speaking the language of the test at home, the principal-reported share of economically disadvantaged students, the share speaking the language of the test, community size, and log school size.

Items were coded by construct: for safety and order measures, higher scores indicated a safer or more orderly school; for belonging and achievement, higher scores indicated a more favorable outcome; and for peer victimization, classroom disorder, and absenteeism, higher scores indicated a less favorable outcome. Consistent with this coding, the fitted slopes were negative for the three adverse outcomes. The safety and order predictors and all outcomes were standardized within the education system, so that cross-national differences in response style and composition could not account for the results. Item wording, response options, orientation after recoding, and aggregation rules are summarized in Table S4. Within-system standardization also rendered the two response-surface axes commensurate [47].

### 2.3. Split-Half Aggregation

Within each school, students were randomly divided into two halves. Half A supplied the child predictor; half B supplied the four child-reported outcomes. No child supplied both the child-reported predictor and a child-reported outcome, and the belonging outcome used items disjoint from the predictor. Achievement was measured on all sampled students, so that for the achievement models the predictor half was not disjoint from the students contributing the outcome; a sensitivity analysis using achievement computed on split-half B alone is reported below. The consequence is that the child predictor rested on a median of 14 students; at that size, the single-item child aggregate (rater-sampling reliability ≈ 0.41; Table S6) was less reliable than the single principal report but more reliable than the two-teacher aggregate (≈0.31), so the design was conservative for the child–principal comparison specifically rather than for both adult comparisons.

A single split would make every estimate depend on one arbitrary partition of the students. The split was therefore drawn 50 times independently, each model was estimated once per split, and the estimates were combined as described below, so that the variability induced by splitting was incorporated into the reported standard errors rather than left unrepresented.

### 2.4. Handling of the Adult Reports and Missing Data

Teacher and principal reports were entered into the primary analyses separately. Where an adult composite was reported as a secondary summary, both an available-item version (averaging whichever adult report was present) and a complete-case version (restricted to schools with both) were computed. Of 12,955 schools, 88.0% had both adult reports, 6.8% a teacher report only, 2.9% a principal report only, and 2.3% neither. The available-item rule accounts for composite-based samples exceeding the principal-report sample; Table S7 shows that the two rules yield the same substantive conclusion. All models used complete cases on the variables they contained, so analytic *n* varied across outcomes.

### 2.5. Analytic Strategy

#### 2.5.1. Reliability, Decomposed

Three quantities were reported separately rather than combined. Internal consistency was measured using the coefficient alpha computed at the level of the individual respondent within each system. Rater-sampling reliability of the school aggregate was estimated in two ways: a model-based ICC(2) from a one-way random-effects decomposition [35], and directly, by randomly splitting raters within schools, correlating the two half-aggregates, and applying Spearman–Brown. For principals, no rater-sampling component was estimable, because a single principal per school constituted the population of currently serving principals. This concerns one variance component only and does not imply that the principal score is free of error; item-, occasion-, and role-specific errors remain. A reliability of 1.00 was therefore not assigned to the principal report. It is observed that internal consistency (α = 0.854 for safety, 0.773 for order) was used as its residual reliability wherever a correction was applied, with 1.00 reported only as an explicit upper bound and 0.80 and 0.70 as lower bounds representing unmodelled occasion and role error. Temporal reliability was not identified in single-occasion data and was not estimated for any informant.

Accordingly, disattenuated correlations were reported as a sensitivity analysis under classical-error assumptions rather than as point corrections, and no comparison was described as insensitive to reliability. Because the three informant predictors differed substantially in rater-sampling reliability, the relative-importance shares reported below were themselves differentially attenuated, with the teachers’ share a lower bound. A reliability-bounded version of the decomposition, in which each informant’s contribution was corrected for its own predictor unreliability under classical assumptions, is therefore reported alongside the observed decomposition (Table S8). Two features render this bound informative. First, each informant’s predictor is the same variable in every outcome model, so its reliability is constant across outcomes; predictor unreliability can therefore shift the level of a given informant’s share but cannot itself generate a between-outcome difference, which is the quantity at issue in the domain-specificity claim. Second, because the three informant scores are nearly orthogonal at the school level, each informant’s incremental contribution is close to its squared zero-order association with the outcome, so dividing each share by that informant’s reliability and renormalizing closely approximates a full errors-in-variables refit. The result is reported as a bound rather than as an estimate, because classical-error assumptions are untenable for aggregates resting on very few raters.

#### 2.5.2. Ceiling Effects

Four estimators were compared: the ordinary Pearson correlation; the correlation on schools below the adult scale maximum; a censored-normal maximum likelihood correlation that modeled the adult report as a normally distributed latent variable right-censored at the maximum, and therefore recovered the correlation that truncation would have otherwise concealed; and the Spearman rank correlation.

#### 2.5.3. Measurement Invariance

Alignment optimization [41] was applied within each informant scale across systems, with a parameter flagged as noninvariant when its aligned value deviated from the cross-system median by more than 0.20 (loadings) or 0.30 (intercepts), against the conventional 25% tolerance [43]. Alignment operates within informants and provides no evidence about comparability across informants; three-item blocks are just identified, so their configural fit is not testable.

#### 2.5.4. Multitrait–Multi-Informant Models

Five confirmatory models were fitted to the 18 school-mean items, including a correlated trait–correlated method minus one specification with the child as the reference informant [48,49] and a six-factor informant × trait model permitting a Campbell and Fiske [50] reading.

#### 2.5.5. Response Surface Analysis and the Three-Predictor Model

For each outcome *Y* and each adult informant *A*,

Y = b_1_C + b_2_A + b_3_C^2^ + b_4_CA + b_5_A^2^ + γ′**X** + system fixed effects + e,

with surface parameters a_1_ = b_1_ + b_2_, a_2_ = b_3_ + b_4_ + b_5_, a_3_ = b_1_ − b_2_, and a_4_ = b_3_ − b_4_ + b_5_ [51,52,53]. Response surface analysis was reported as a secondary analysis of directional discrepancy rather than as a congruence test. A nonzero a_3_ indicates that the direction of disagreement is associated with the outcome; a negative a_4_ would be the strict discrepancy-as-risk pattern. Interchangeability was not inferred from a significant a_1_ with null a_3_ and a_4_, because congruence conclusions required conditions on the entire surface that a subset of parameters could not establish, and the informant measures employed here were not parallel instruments [47]. All five polynomial coefficients and all four surface parameters for every model are reported in Table S9. To place informants on a common scale, the coefficient share was computed as |b_2_|/(|b_1_| + |b_2_|) in the pairwise models, and each |b|, as a proportion of the summed |b| in the three-predictor model, 0.50 and 0.33 respectively, indicated an equal contribution. This index compares standardized slope magnitudes. It is not a partition of variance: it ignores the correlations among the informant scores, it is insensitive to sign, and it can move with predictor reliability, so a share of 0.81 should not be interpreted as 81% of the information.

The coefficient share was therefore accompanied by an exact variance decomposition. The three-predictor model regressed each outcome on the child, teacher, and principal safety scores simultaneously: Y = β_1_·child + β_2_·teacher + β_3_·principal + γ′X + system fixed effects + e, using linear informant terms only (no quadratic or interaction terms). On this model the LMG (Lindeman–Merenda–Gold) relative-importance measure [54] averaged each predictor’s incremental R^2^ over all 3! = 6 orders of entry and so partitioned variance into non-negative shares. Two interpretive considerations apply. The education-system fixed effects and the covariate were retained in every submodel and did not enter the decomposition, so that LMG partitioned the informants’ joint incremental R^2^ over and above these terms rather than the total variance of the outcome. Because a share is a ratio, between-outcome differences were tested directly on the bootstrap distribution of the difference rather than by the inspection of interval overlap. LMG shares were reported alongside the coefficient shares, together with the total variance explained by the three informant scores, so that relative and absolute magnitudes could both be evaluated.

#### 2.5.6. Combining Splits and Plausible Values

Plausible values are proper multiple imputations and are combined by the standard rule. Meanwhile, random partitions of the same observed students are not; the same students recur across splits, no missing-data posterior is involved, and the partition is a randomization device selected by the analyst rather than a source of missing information. Splits were therefore not treated as imputation completions.

The point estimate was the average over 50 independent splits, which removed dependence on any single partition. The five plausible values were not averaged before resampling. Each plausible value was carried separately through the entire procedure, yielding a split-averaged estimate Q_m and a bootstrap sampling variance U_m for each; the three components were then combined in the standard manner—$\bar{Q}$ = mean(Q_m), Ū = mean(U_m), B = var(Q_m), and T = Ū + (1 + 1/M)B—with Rubin degrees of freedom, so that the between-plausible-value component was added explicitly rather than being absorbed by averaging. Outcomes with no plausible values used the percentile bootstrap directly. All uncertainty was derived from an outer bootstrap that resampled the 63 education systems with replacement and re-executed the entire split-and-plausible-value procedure within each of 1000 replicates; percentile intervals are reported. Education systems are the exchangeable unit for a cross-system estimand. Between-split variability was reported descriptively as the Monte Carlo standard error of the split average; across all reported quantities, it did not exceed 0.009, an order of magnitude below the bootstrap standard errors, indicating that 50 splits were sufficient. The bootstrap was sufficiently large that resampling noise in the reported interval endpoints was negligible: recomputation of the percentiles on resamples of the 1000 replicates moved the endpoints of the child-share intervals by a Monte Carlo standard error of at most 0.010 (Table S10).

Because repeated sample splitting combined with plausible values is not a standard procedure, its calibration was verified by simulation rather than assumed. A simulation with a finite population of 300 education systems, with schools nested within them and students nested within schools, and a known estimand compared three interval procedures over 225 replications (Table S11). Treating splits as imputation completions covered the estimand in 100% of replications with intervals approximately 39% wider than required; a single split with cluster-robust errors covered 93.3%; the procedure adopted here covered 96.0% and was the narrowest of the three.

#### 2.5.7. Weighting and Inference

Because education systems contributed different numbers of schools, the results are reported under four schemes: TIMSS school sampling weights normalized within systems; equal system weights; unweighted pooled school-level estimation; and system-specific estimates combined by restricted maximum likelihood random-effects meta-analysis with Hartung–Knapp confidence intervals and prediction intervals [55,56], with between-system heterogeneity (τ, I^2^) reported. Hartung–Knapp was used in preference to DerSimonian–Laird because it maintained nominal coverage that was substantially better when the number of studies—here, education systems—was moderate. Bootstrap confidence intervals resampled education systems, thereby treating the system as the exchangeable unit; this is appropriate because sampling, translation, and administration decisions operate at that level.

#### 2.5.8. Shared-Rater Inflation

Rater count was held constant. Both predictors were half-sample aggregates; the conditions differed only in whether the predictor and outcome were derived from the same students. The shared-rater condition used half A → half A and half B → half B; the independent condition used half A → half B and half B → half A. Both directions were averaged and the procedure was replicated over 200 random within-school splits.

#### 2.5.9. Software, Estimators and Settings

All analyses were run in Python 3.10.12 with numpy 2.2.6, scipy 1.15.3, pandas 2.3.3, scikit-learn 1.7.2, matplotlib 3.10.9, pyreadstat 1.3.5 (SPSS file input) and semopy 2.3.11 (structural equation models). The multitrait–multi-informant models were estimated by maximum likelihood on the covariance matrix of the 18 school-mean items, with latent variances fixed to one for identification, listwise deletion applied to the 11,084 schools with complete item-level data, and default semopy convergence criteria; models were fitted unweighted at the school level, and their fit statistics were descriptive rather than the basis of any inference. Alignment optimization was implemented directly following Asparouhov and Muthén, minimizing the component loss function f(x) = (x^2^ + ε)^¼ with ε = 0.01 over configural one-factor solutions estimated separately in each education system; the 0.20 and 0.30 flagging thresholds for loadings and intercepts were the conventions used in that literature and were applied here for comparability, with the aligned parameter dispersions reported alongside the flag rates in Table S3 so that readers could impose a different standard. Student aggregates were weighted by the total student weight within schools; teacher aggregates were unweighted means of responding teachers; achievement school means were weighted by the total student weight and computed on all sampled students, because achievement was not drawn from the child questionnaire and could not share raters with a child report. Response surfaces were estimated by ordinary least squares with education-system fixed effects. All reported intervals for the questionnaire outcomes in Table 3 and Table 4 are percentile intervals from 1000 bootstrap resamples of education systems. For the achievement outcomes, each plausible value was analyzed separately, the bootstrap sampling variance was estimated within each plausible value, and the estimates and variances were combined by Rubin’s rules, so achievement intervals additionally incorporated plausible-value uncertainty. The Hartung–Knapp and prediction intervals in Table 4 come from the restricted maximum likelihood meta-analysis. The latent profile models used diagonal covariance structures with 20 random starts, the best replicated log-likelihood retained, default expectation–maximization convergence tolerance, and 50 bootstrap replications for the bootstrapped likelihood ratio test; with 50 replications, the smallest attainable *p* value was 0.02, which accounted for the recurrence of that value in Table S12.

### 2.6. Transparency, Openness and Use of Generative AI

The latent profile analysis tested a typology specified before the model was estimated; all other analyses were exploratory in the sense of Wagenmakers et al. [57], and every specification estimated is reported here or in the Supplementary Materials. The TIMSS 2023 database is publicly available, and the full analysis code reproducing every result, table and figure is deposited openly. Generative artificial intelligence tools were not used in the study design, data collection, analysis, or interpretation, or in the generation of scientific content, data, or figures.

## 3. Results

### 3.1. Distributions of Informant Reports and Ceiling Concentration

**Figure 1:**
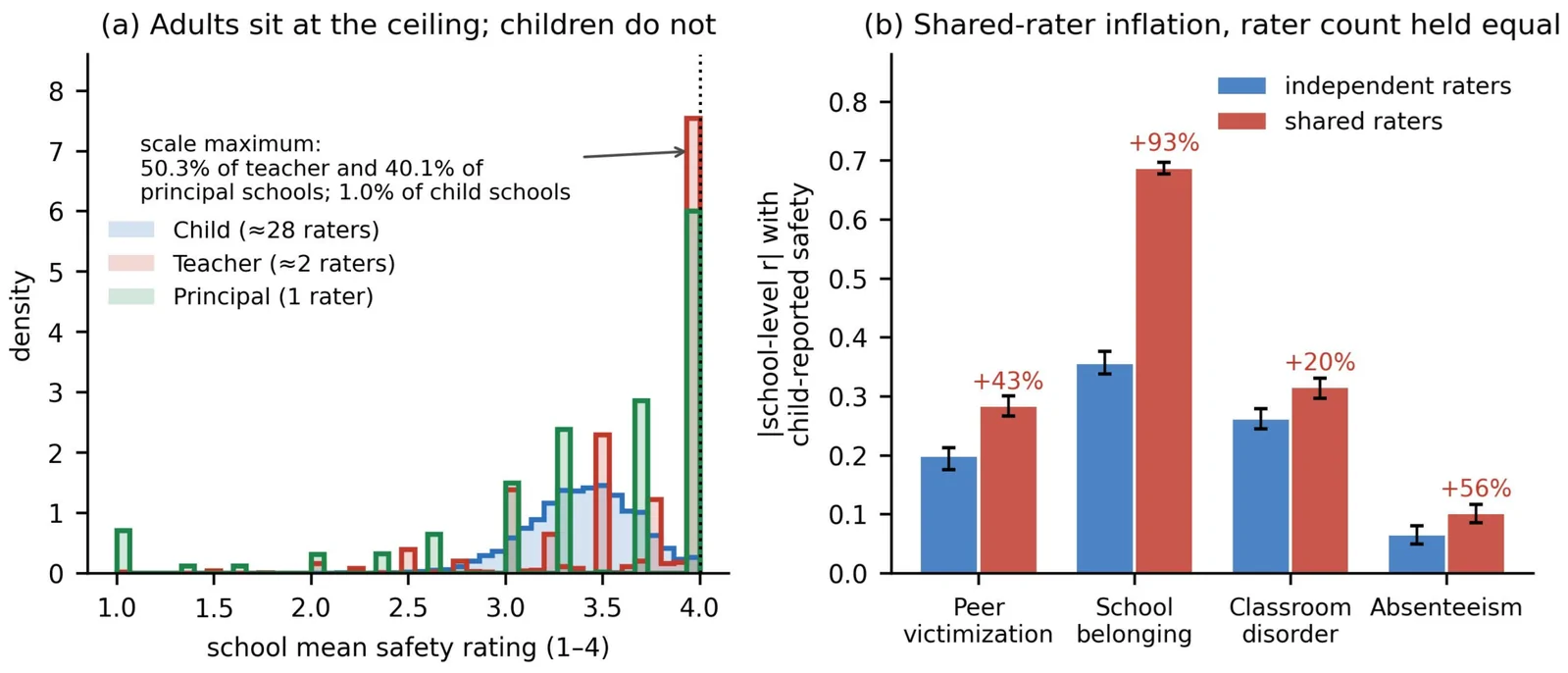
Rating distributions by informant, and the effect of shared raters. (**a**) School-level distributions of child, teacher and principal safety reports on the 1–4 scale; half of teacher-reporting schools and two fifths of principal-reporting schools were at the absolute maximum, against 1.0% of child-reporting schools. The child distribution is shown for full-sample school means (median ≈28 respondents); the primary child predictor, instead, was based on a median of ≈14 respondents (split-half A). (**b**) Absolute school-level association between child-reported safety and each child-reported outcome when predictor and outcome come from independent halves of the students versus from the same half; rater count was held equal across conditions, so that the difference isolates shared-rater covariance. Error bars span the 2.5th to 97.5th percentiles across 200 random within-school splits.

Mean school safety was *M* = 3.40 (*SD* = 0.27) from children, 3.65 (0.48) from teachers and 3.41 (0.77) from principals on a 1–4 scale. The distributions differed considerably more than the means (Figure 1a). Among schools with a teacher report, 50.3% were at the absolute ceiling, with every item endorsed maximally, as were 40.1% of schools with a principal report, against 1.0% of child-reporting schools. For approximately half of the schools, therefore, the adult reports exhibited no residual variance.

### 3.2. Reliability Components

Table 2 reports the three components separately. Internal consistency was 0.64 for the three-item child scale, 0.78 for the two-item teacher scale and 0.85 for the three-item principal safety scale. Rater-sampling reliability of the school aggregate was 0.63 (ICC(2)) and 0.61 (direct split-half) for child safety—close agreement indicating that the model-based estimate was not an artifact of the one-way random-effects assumption. For teachers, it was weaker still once records were reduced to unique teachers: a single teacher’s report correlated 0.202 with an independent teacher’s report in the same school, giving an aggregate rater-sampling reliability of 0.314 for the 1.9 unique teachers actually available. This value of 0.314 was the direct empirical split-half aggregate reliability, which reflected the variable number of responding teachers per school and the weighting; a nominal Spearman–Brown step-up of the rounded 0.202 to 1.9 raters gives ≈0.33. A school-level teacher safety score in TIMSS Grade 4 was therefore empirically close to a single-rater report. The principal score carried no rater-sampling error by construction, but its internal consistency was 0.85 and its temporal stability was unknown; that internal consistency, rather than 1.00, was used wherever a correction was applied.

### 3.3. RQ1: Within-Informant Comparability

Across all six informant-by-trait blocks, 0.0% to 5.6% of loading and intercept parameters were appreciably noninvariant, far inside the 25% tolerance and below 2% in five of six blocks (Table S3). The sixth block, child-reported classroom order, behaved comparably (1.2% noninvariant across 62 systems; Table S13), although its one-factor configural fit was poor on the RMSEA (median CFI = 0.876, RMSEA = 0.319); the block is short and spans two subject lessons, which inflated the RMSEA at low degrees of freedom. The RQ1 conclusion is therefore weaker for this block, and modest for teacher safety and security (median CFI = 0.860); both are interpreted narrowly. Alignment reduced the cross-system dispersion of loadings from 0.076–0.238 to 0.040–0.082 and of intercepts from 0.189–0.300 to 0.057–0.155, and the aligned factor means correlated 0.94 to 0.999 with simple system means. The one block with appreciable noninvariance was principal order problems (5.6%), driven by the lateness and absenteeism intercepts; it was not used as a predictor.

These results warrant a narrow interpretation. Within-informant alignment detected relatively little parameter noninvariance, although several short blocks were identified, and alignment did not establish equivalence across informants. The published TIMSS teacher and principal composites were approximately invariant when constrained to a one-factor model (0.8% and 5.3% noninvariant) but fitted that model poorly (median CFI = 0.793 and 0.882; RMSEA = 0.234 and 0.200), which constitutes evidence for the two-dimensional safety-versus-order structure adopted here. Replacing item means with configural factor scores left every substantive parameter essentially unchanged (Table S14).

### 3.4. RQ2: Cross-Informant Convergence and Its Rival Explanations

Median school-level correlations across the 63 systems were *r* = 0.078 (child–teacher), 0.092 (child–principal) and 0.116 (teacher–principal); pooled within-system estimates were 0.082 and 0.094.

The ceiling-effect sensitivity estimators did not materially alter the result. The median share of schools at the adult ceiling was 47.5% (teacher) and 40.5% (principal), so the potential for range restriction was substantial (Table S15, Figure S1). Restriction to non-ceiling schools lowered median convergence to 0.042 and 0.030. The censored-normal estimator recovered little additional convergence, returning 0.064 and 0.081—indistinguishable from, and marginally smaller than, the uncorrected values—and the magnitude of the correction did not increase with ceiling severity. Both checks were sensitivity analyses rather than reconstructions of what the ceiling concealed: school-level adult aggregates are bounded, discrete and heavily concentrated at the maximum, so a censored-normal latent distribution is an assumption, and the non-ceiling subsample is a selected population rather than an unbiased correction. Under that estimator, child–teacher convergence was below 0.20 in 60 of 63 systems and child–principal in 56 of 63. Under these assumptions the correction was small, because the reports were sufficiently close to orthogonal that the truncated region contained no more signals than the untruncated region.

**Figure 2:**
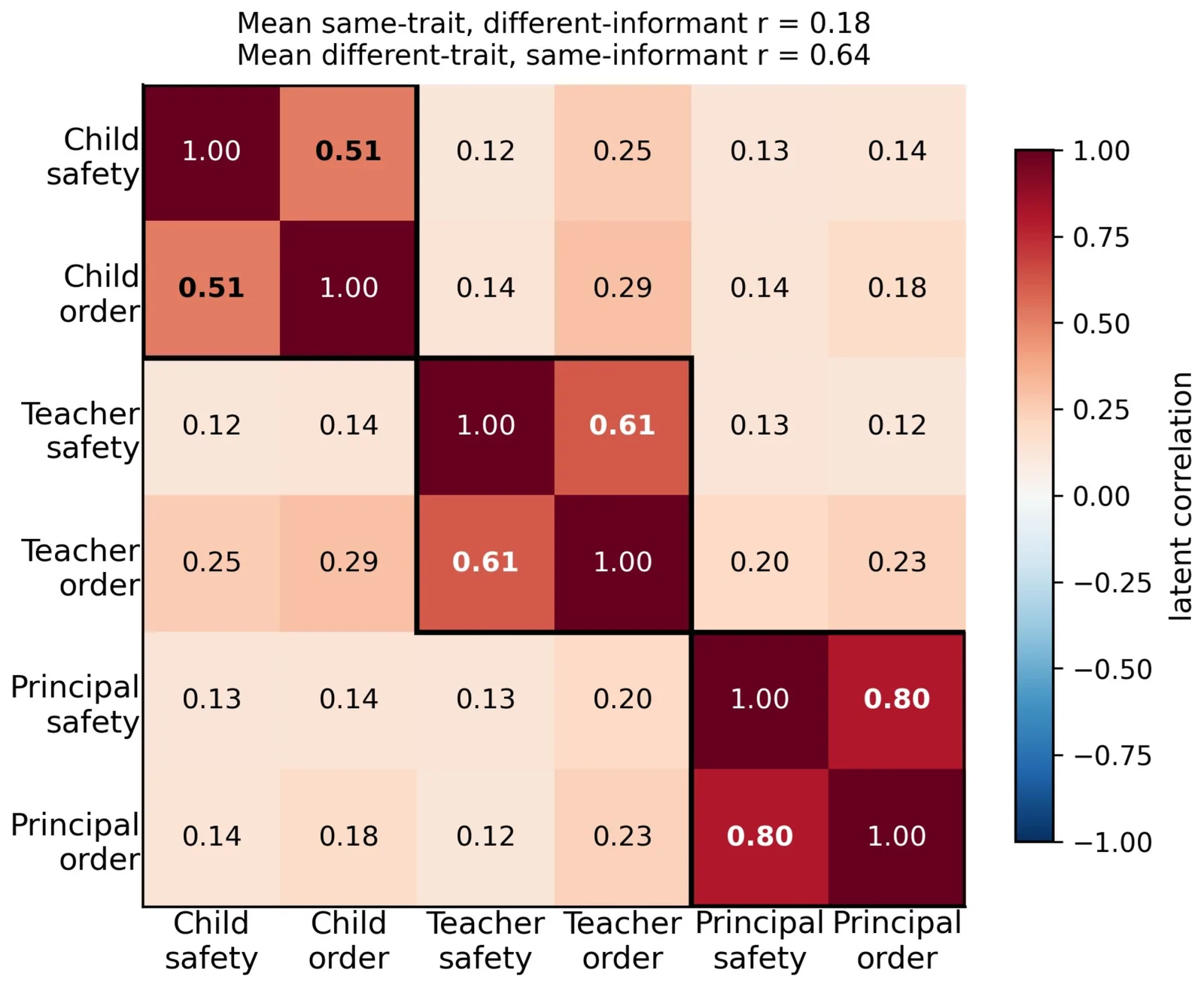
Latent multitrait–multi-informant matrix, *n* = 11,084 schools in 63 education systems. Boxed blocks are within informants; the bolded off-diagonal entry in each box is that informant’s different-trait, same-informant correlation. Same-trait, different-informant coefficients lie outside the boxes and are uniformly smaller. Teacher and principal factors correlated only 0.187 with one another; the two adult informants were therefore analyzed separately rather than averaged.

The six-factor latent model fitted acceptably, χ^2^(120) = 5590.6, CFI = 0.952, RMSEA = 0.064, far better than a single common factor (CFI = 0.381) or two trait factors (CFI = 0.439). The mean same-trait, different-informant correlation was 0.179 against a mean different-trait, same-informant correlation of 0.636, a ratio of 3.56 (Figure 2, Table S16). Correcting for aggregation unreliability and assigning the principal its observed internal consistency of 0.854 reduced the ratio to 3.00. Treating the principal score as error-free—an upper bound that is reported but not adopted—yielded 2.85, and lower bounds of 0.80 and 0.70 yielded 3.02 and 2.46, so that the ratio spanned 2.46 to 3.02 across the range (Table S5). One to two entries exceeded unity in every case. These inadmissible values indicate that the classical-error model is untenable for aggregates based on very few raters, and all disattenuated values were therefore treated as sensitivity analyses rather than as estimates. The substantive conclusion does not depend on them: under every assumption examined, informant identity remained substantially more informative than the construct rated.

With the child as the reference informant, 66.8% of child indicator variance was loaded on the trait factors against 2.2% for teachers and 0.6% for principals, with 51.8% and 53.6% of adult indicator variance being informant specific. The teacher and principal informant factors correlated by only 0.187: the two adult informants were nearly as dissimilar from each other as each was from the children, and they were therefore not averaged in the analyses that follow.

### 3.5. RQ3: Domain Specificity of Criterion-Related Informant Validity

**Figure 3:**
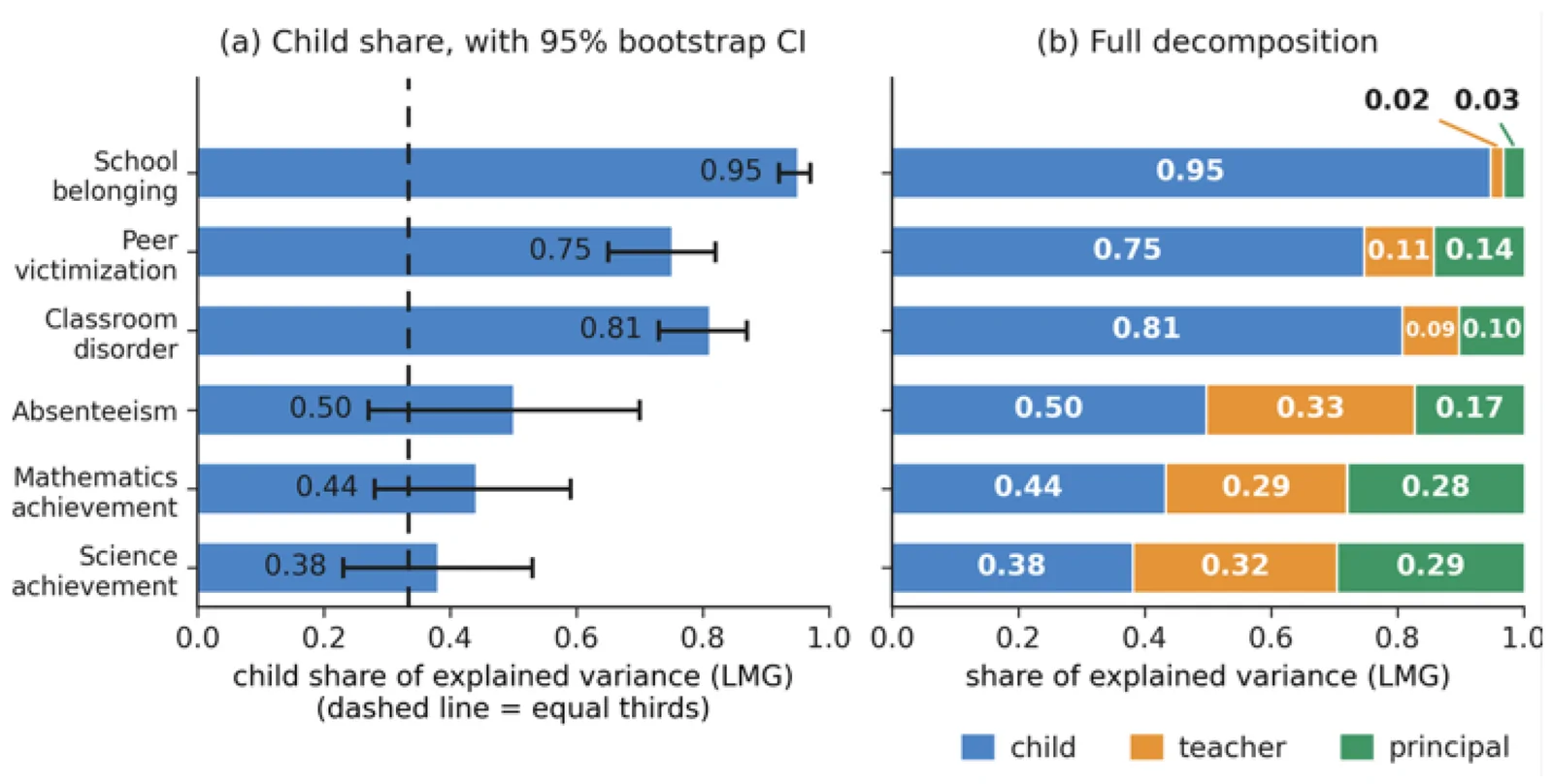
**Domain specificity of criterion-related informant validity.** (**a**) Share of the variance the three informant scores jointly explain that is attributable to the child report, by the exact LMG decomposition, with 95% percentile intervals from 1000 bootstrap resamples of education systems (for achievement, plausible-value uncertainty is additionally incorporated by Rubin’s rules); the dashed line marks equal thirds. (**b**) The full decomposition across child, teacher and principal. The child predictor is the referent-matched single item I feel safe at school. Point estimates average 50 random within-school splits and, for achievement, five plausible values. The belonging and achievement intervals do not overlap.

The three-predictor LMG decomposition in Panel C of Table 3 was the primary analysis for this question, because it is the only one of the two reported indices that partitions variance; Panels A and B present the pairwise response surfaces as a secondary description of directional discrepancy, and the full set of polynomial coefficients and surface parameters is given in Table S9, with the fitted surfaces plotted in Figure S2, rather than in the main text. Figure 3 summarizes the decomposition.

Child with teacher (Table 3, Panel A). For school belonging, the child slope was b_1_ = 0.378 against b_2_ = 0.046, giving a_3_ = 0.332, 95% CI [0.290, 0.377]; for peer victimization, b_1_ = −0.222 against b_2_ = −0.085 and a_3_ = −0.138 [−0.192, −0.090]; and for classroom disorder, a_3_ = −0.209 [−0.270, −0.154]. For mathematics achievement, the two contributed almost identically (b_1_ = 0.136, b_2_ = 0.144) and a_3_ was indistinguishable from zero, as it was for absenteeism. Adult coefficients were small relative to the child coefficients but were not zero: for belonging and victimization, the teacher intervals excluded zero.

Child with principal (Table 3, Panel B). The same pattern held: belonging a_3_ = 0.292 [0.241, 0.348], victimization a_3_ = −0.091 [−0.135, −0.046], disorder a_3_ = −0.156 [−0.212, −0.099], mathematics a_3_ = −0.035 (ns), and absenteeism a_3_ = −0.003 (ns). Principal coefficients were larger in magnitude than teacher coefficients for five of the six outcomes (belonging b_2_ = 0.080, victimization b_2_ = −0.127); for absenteeism, the two were similar (−0.071 vs. −0.075). The adult coefficient share rose from 0.18 (belonging) through 0.32 (disorder) and 0.37 (victimization) to 0.49 (absenteeism) and 0.56 (mathematics).

Three-predictor model (Table 3, Panel C; Figure 3). With all three informants retained, the variance explained by the informants was divided very unevenly, and the LMG decomposition was more discriminating than the coefficient share. The child report accounted for 95% of the informant-explained variance in school belonging, 95% CI [92%, 97%], against 2% for teachers and 3% for principals; 75% [65%, 82%] for peer victimization, with 11% and 14% for the two adults; and 81% [73%, 87%] for classroom disorder. For absenteeism the shares were 50% [27%, 70%], 33% and 17%, and for mathematics achievement 44% [28%, 59%], 29% and 28%—close to equal thirds. Science achievement was 38% [23%, 53%], 32% and 29%. Rather than inferring the gradient from overlapping intervals, the differences were tested directly on the bootstrap distribution: the child share was 0.516 higher for belonging than for mathematics, 95% CI [0.376, 0.686], and 0.309 higher for victimization than for mathematics [0.203, 0.425]. Neither difference reversed signs in any of the 1000 bootstrap replicates, so both *p* = 0.001, the smallest attainable value under the plus-one rule with 1000 two-sided bootstrap replicates. The coefficient shares yielded the same ordering across a compressed range (child 0.82, 0.58, 0.63, 0.43, 0.39 and 0.36 respectively).

The absolute magnitudes qualify the interpretation of these proportions. The three informant scores jointly explained 13.2% of the between-school variance in belonging, 8.2% in classroom disorder, 5.5% in victimization, 4.5% in mathematics and 1.1% in absenteeism, after system fixed effects. The claim concerns the division of explainable variance among informants, not the proportion of between-school variation in school functioning that informant reports account for.

**Reliability-bounded decomposition.** The three informant predictors differed substantially in reliability, with 0.610 for the child aggregate, 0.314 for the teacher aggregate and 0.854 for the single principal report. Consequently, the observed shares were differentially attenuated and the teacher share constituted a lower bound. Correcting each informant’s contribution for its own predictor unreliability (Table S8) raised the teacher share substantially where it was largest, from 29% to 47% for mathematics and from 32% to 51% for science, while leaving it small for belonging (2% to 4%). The correction did not attenuate the domain gradient but widened it. The child share fell from 95% to 94% for belonging but from 44% to 37% for mathematics, so that the belonging-versus-mathematics difference increased from 51 to 57 percentage points and the victimization-versus-mathematics difference from 31 to 34 points. Setting the principal’s residual reliability at the lower bound of 0.70 or at the upper bound of 1.00 rather than at its observed alpha changed no corrected share by more than three percentage points. The insensitivity of the gradient to this correction is structural rather than empirical: each informant’s predictor is the same variable in all six outcome models, so predictor unreliability shifts the level of that informant’s share but cannot produce a difference between outcomes. Two cautions apply. Classical disattenuation is untenable at a reliability of 0.314; the corrected teacher values therefore indicate that the teacher’s true contribution to the achievement outcomes was appreciably larger than the observed decomposition implies, without estimating its magnitude; and the correction addresses attenuation in the predictors only.

**The child–principal comparison as primary evidence.** Because the principal report carried no rater-sampling error by construction, the child–principal contrast was the less attenuated of the two child–adult comparisons and the more direct test of the domain claim; it was accordingly treated as the primary evidence and the child–teacher contrast as corroborating. It exhibited the same gradient without reliability correction: the principal share rose from 3% for belonging to 14% for victimization, 17% for absenteeism, 28% for mathematics and 29% for science, and the pairwise incongruence slope fell from a_3_ = 0.292 for belonging to a value indistinguishable from zero for mathematics and absenteeism.

This constitutes the principal result of the study, and the pattern across outcomes is more informative than any single coefficient. The child report did not predominate across all outcomes: it predominated for outcomes describing children’s experience of school and approached parity with the adult reports for the externally assessed outcome. This parity does not represent a limitation of the adult reports but the least confounded comparison in the design, since achievement was externally assessed and neither shared raters nor shared perspective could operate on the outcome side.

**Robustness.** Every conclusion survived the referent-matched single item (used throughout Table 3), the three-item scale, socioeconomic and composition controls, and the exclusion of the four benchmarking entities nested within a participating country (Abu Dhabi, Dubai, and Sharjah within the United Arab Emirates, and Quebec within Canada; Table S1). Table 4 reports a_3_ under four weighting and inference schemes. Two design sensitivity analyses were conducted. First, because achievement was measured on all sampled students and therefore overlapped the students supplying the child predictor, the achievement models were re-run with achievement computed on split-half B alone, so that predictor and outcome rested on disjoint children. The child coefficient fell slightly (mathematics b_1_ = 0.136 to 0.121; science 0.132 to 0.118), the adult coefficient fell comparably, a_3_ remained negligible (−0.008 to −0.015 for teachers), and the child LMG share changed from 44% to 40% for mathematics and from 38% to 36% for science. Mechanical overlap therefore inflated the child share by at most approximately four percentage points and could not account for the gradient. Second, resampling schools within systems as well as systems, as a check on within-system design variance, left every interval essentially unchanged (belonging a_3_ [0.283, 0.381] against [0.290, 0.377]; victimization [−0.199, −0.077] against [−0.192, −0.090]) on the same 1000 replicates.

Under TIMSS school weights, equal system weights, unweighted pooling and a random-effects meta-analysis—all averaged over the same 50 splits and five plausible values—belonging a_3_ ranged from 0.262 to 0.331 and victimization a_3_ from −0.054 to −0.137, while achievement a_3_ remained small under every scheme. Combining the system-specific slopes (Table S17) by restricted maximum likelihood with Hartung–Knapp intervals, belonging (a_3_ = 0.326, 95% CI [0.293, 0.360] for teachers; 0.276 [0.237, 0.315] for principals) and victimization (−0.133 [−0.175, −0.091]; −0.068 [−0.107, −0.029]) excluded zero, while absenteeism did not; the principal-model summary intervals for mathematics and science narrowly excluded zero (−0.068 [−0.134, −0.003] and −0.079 [−0.144, −0.014]), although their prediction intervals crossed zero widely. Between-system heterogeneity ranged from negligible to substantial (τ = 0.000 to 0.193; I^2^ = 0% to 62%), and the prediction intervals in Table 4 are wide: in a new education system, the belonging slope would be expected to lie between 0.17 and 0.48 for teachers. The central ordering was not weighting-dependent, although system-level variation was substantial and warranted further investigation.

### 3.6. Prespecified Check: Latent Profile Analysis of Informant Configurations

Latent profile analysis provided no support for a typology (Table S12). The Bayesian information criterion (BIC) declined monotonically with no elbow, entropy peaked at 0.697 for two classes and fell thereafter, the smallest class fell below 7% by five classes, and both likelihood ratio tests rejected the k − 1 model at every step computed with no stopping point—the pattern expected when a continuous distribution is partitioned rather than a latent class structure recovered [58,59]. Critically, profile membership added a ΔR^2^ of no more than 0.0014 over the continuous informant scores for any outcome. Schools differed continuously in the degree of informant divergence rather than falling into discrete types.

### 3.7. Shared-Rater Inflation, with Rater Count Held Equal

With both predictors based on half samples, so that the conditions differed only in whether the predictor and outcome were derived from the same students, sharing raters inflated the school-level association by 43% for peer victimization (from |r| = 0.198 to 0.282), 93% for school belonging (0.355 to 0.686), 20% for classroom disorder (0.261 to 0.314), and 56% for absenteeism (0.064 to 0.100), with intervals across 200 random splits given in Table S18 and Figure 1b. This estimate isolates shared-rater covariance; a comparison against a full-sample predictor would confound it with the higher reliability of that predictor and should not be interpreted as shared-rater inflation.

## 4. Discussion

In 63 education systems, children, teachers and principals in the same schools produced descriptions of school safety that were close to statistically independent. Neither examined sensitivity analysis materially accounted for it: under the ceiling-effect sensitivity estimators, the convergence estimates were essentially unchanged, and differential aggregation unreliability narrowed but did not remove the trait-versus-informant contrast under any assumption about principal reliability examined here. The novel contribution is not the demonstration that informants disagree but the characterization of what that disagreement is informative about.

### 4.1. Domain Specificity of Criterion-Related Informant Validity

The principal result is the gradient presented in Figure 3. As the outcome moved from school belonging to peer victimization and classroom disorder, then to absenteeism and achievement, the share of explained variance attributable to the two adult informants rose from approximately one twentieth to more than half. The coefficient share and the LMG variance decomposition ordered the outcomes identically, which supports the gradient interpretation; the LMG values are preferred for reporting, because only they partition variance. The claim was falsifiable in a specific respect: had the child report predominated for achievement as it did for belonging, the finding would have reduced to a statement about informant identity alone. This was not observed.

This is more limited than the conclusion that adult reports are invalid, and the achievement result is where the limit is clearest. Achievement is the only outcome in the design that is externally assessed rather than reported by any of the three informants, so it is the one comparison in which no shared-rater and no shared-perspective mechanism can operate. It is therefore the evidence least susceptible to method effects, and it demonstrates parity: adult-reported safety predicted achievement approximately as well as child-reported safety did, and predicted absenteeism at least as well. This parity is interpreted as a substantive strength of the adult reports rather than solely as the boundary of the child report’s advantage. On the outcome least vulnerable to the methodological objections that can be raised against the rest of the design, adults reported the school as a learning environment as informatively as children did, which indicates that adult reports index genuine institutional properties rather than reflecting ceiling effects or accountability incentives. The defensible conclusion is correspondingly narrower than a claim of adult invalidity and sufficient: adult reports should not be treated as interchangeable substitutes for children’s reports of experienced safety.

Explicit specification of what this gradient does and does not establish is required, because informant and construct coverages are confounded by the instrument. The adult items ask about the school as an institution, i.e., the teacher’s own safety, the school’s security policies, the frequency of disciplinary incidents, whereas the child items ask about the child’s own experience of being at school. The gradient is therefore in part a statement about what the available adult items measure and not only about what adults as informants are positioned to know, and the two readings should not be conflated. On the narrow, construct-coverage reading, the finding is that institutional-climate items—the items administered to adults in school-health systems—do not capture the experiential outcomes that surveillance most requires, and surveying additional adults with the same items would not remedy this. On the broader, informant-validity reading, adults occupy a position from which experienced safety is not directly observable, so no adult instrument would fully close it. This design cannot separate the two, because referent, item content and level of observation all vary alongside informant; the internal multitrait–multi-informant contrast and the referent-matched single item constrain but do not eliminate this ambiguity. What can be established is that the two interpretations share the operative implication, and it is this implication that is claimed here: the adult-reported instruments currently in use cannot substitute for children’s reports of experienced safety. The evidence that at least part of the effect is positional rather than purely instrumental is suggestive rather than decisive—it coheres with the finding that classroom-level factors track teachers’ perceptions while school-level factors track students’ [24], with the multilevel multi-informant structure of Konold et al. [25], and with the phenomenology of victimization [15], much of which occurs in forms not observable to supervising adults—and the conclusion does not rest on it.

### 4.2. The Shared-Perspective Account

A competing account requires explicit consideration. Four of the six outcomes were child-reported. Split-half aggregation removes shared raters but not shared perspectives: students in the same school may share a mode of construing that school, which persists across any two random halves. Under this account, the child report predominated for child-reported outcomes not because children had privileged access but because a school-level child method factor contributed to both predictor and outcome.

Three considerations are worth attention. The simplest version of the account—a school-level child method factor of roughly uniform strength—predicts a uniform child advantage across the four child-reported outcomes, and there is none: the child share ranges from 95% for belonging to 50% for absenteeism. That observation constitutes weaker evidence than it may appear, however, because a method factor need not be uniform. A shared mode of construal would plausibly load more heavily on evaluative outcomes such as belonging than on comparatively concrete ones such as days absent, and a method factor graded in that way would reproduce the observed gradient without any privileged access on the children’s part. The second consideration is more robust to that objection: the account predicts no child advantage for the externally assessed outcomes, and there is none, which bounds the method factor to the child-reported side of the design and indicates that it cannot provide a complete account. The third—that the effect sizes were largest for outcomes concerning discrete events rather than global evaluations—is weakly consistent with the same conclusion. Because the first consideration cannot be sustained in its strongest form, the shared-perspective account is treated as partially correct rather than as refuted, and the evidence supports the conclusion that both mechanisms operate. This bears on how the result should be described: the evidence does not support the claim that adult reports are inaccurate with respect to children’s safety. What the data support is that child-reported well-being outcomes were predicted substantially less well by adult reports than by children’s own reports—whether because adults do not observe what children observe or because children’s shared construal is itself a component of what a safety indicator should capture. Under either interpretation, adult-only measurement captured substantially less amount of variance of that construct.

### 4.3. Relation to the Existing Literature

The disattenuated child–adult correlations obtained here were approximately half to two thirds of the 0.28 reported by Achenbach et al. [18] for informants describing the same child. Two features explain the difference: the aggregation is asymmetric, and the referent is an institution rather than a child, which affords far more scope for perspective-dependent construal. The Operations Triad Model [22] frames the interpretation, and four results favor diverging over converging operations: informant-specific variance was large and structured; the discrepancy predicted outcomes directionally rather than symmetrically, a pattern inconsistent with measurement error; the teacher and principal informant factors correlated only 0.187; and each informant’s relative weight varied systematically with outcome domain. This extends the multi-informant validity argument of De Los Reyes et al. [20] from individual to organizational assessment.

The present findings also illustrate why difference scores mislead [23]. Had outcomes been regressed on an adult-minus-child difference score, a discrepancy effect would have been reported and disagreement would have been interpreted as a risk marker. The significant parameter was a_3_, the directional slope, and not a_4_. Disagreement per se was not associated with the outcome; a low child report was.

Finally, the achievement results do not conflict with the literature finding that adult-reported climate predicts attainment [14,60]. The present results are consistent with that finding. The claim made here is narrower: adult reports carried as much information as children’s about the school as a learning environment, and substantially less—though not none—about a child’s experience of threat. At the school level, victimization and attainment may be dissociated, and this dissociation occurred precisely in the schools that adult-informant measurement tracked least well.

### 4.4. Implications for School Health, Well-Being Surveillance and Prevention

The choice of informant propagates directly into four core functions of school-health systems—surveillance, psychosocial-risk identification, preventive intervention, and resource allocation—so the validity results above have concrete public-health consequences. Six recommendations follow, organized around those functions.

Student self-report should be included in school-safety surveillance. Where the target is children’s experienced safety, the child report is not one informant among several; it is the criterion. A short child questionnaire, aggregated across a grade, would provide a school-level indicator that adult reports track only weakly. National health surveillance already treats student-reported connectedness as a core indicator [12], and student-report wellness indices are now used for population monitoring within multi-tiered systems of support [17]; the present results indicate why child self-report cannot be replaced by adult proxy report in these systems.

Adult and child indicators should be reported separately, not averaged. Averaging informants whose reports correlate near zero and whose validity is domain-specific eliminates the only information that distinguishes institutional order from experienced safety. The present results indicate why: the teacher and principal informant factors were correlated by 0.187, so adult report did not constitute a single perspective.

“Institutional safety” and “experienced safety” should receive different labels. Adult-reported safety is a valid measure of school safety as perceived by adults at the institutional level. Designating it as school safety without qualification invites the substitution against which the present results argue.

Approximately 30 student responses support a school-level indicator but not a validated screening instrument. Child aggregates of about 28 respondents reach a rater-sampling reliability near 0.61; two teachers reach 0.31. A single item—I feel safe at school—reproduced the substantive findings, and the questionnaire burden was therefore minimal. The scope of this conclusion should be noted. A reliability of 0.61 supports reporting a school-level indicator with an uncertainty interval; it does not establish a screening instrument. No unsafe-school criterion, sensitivity, specificity, calibration, or held-out classification accuracy was estimated, and none of these quantities can be inferred from a reliability coefficient. Developing and cross-validating a classification rule constitutes a separate study, and the feasibility literature on universal school-based identification and whole-child screening is the appropriate guide for that step [16,61].

Discordance should prompt further enquiry, not automatic classification. Schools do not fall into informant types, and dichotomizing a continuum to produce reporting categories discards information without yielding valid classes [58]. The relevant question is not whether adults and children agree, since they typically do not, but whether child-reported evidence is discordant with the adult account.

Health and counseling services should not rely on principal reports alone to allocate preventive support. On the present estimates, the principal’s report accounted for approximately 3% of the variance that the three informants jointly explained in children’s sense of belonging (LMG share 0.028), which, given a total R^2^ of 0.132, corresponds to approximately 0.4% of the between-school variance within systems. Because schools are the setting in which most children who receive any mental-health service receive it [3], allocation errors at this stage have direct consequences for which children are reached: allocating psychosocial resources on the basis of adult reports alone is unlikely to distinguish schools where adults perceive order and children experience isolation. Equally, whole-school preventive programs [4] can be directed efficiently only if the schools of greatest need are identified from valid inputs.

Between-system heterogeneity in the incongruence slopes ranged from negligible to substantial (I^2^ = 0–62%), and the prediction intervals were wide enough that a new education system could plausibly show an appreciably weaker or stronger child advantage than the summary estimates imply. The ordering—that adult reports carry the least weight where the outcome is experiential—appears generalizable; the magnitude does not, and the optimal combination of informants may vary by context. Systems differ in class size, in the degree to which principals are held accountable for reported safety, in the number of responding teachers available to a school-level aggregate, and in the adequacy of child instruments following translation for 9-year-old respondents; each of these may plausibly alter the balance. The recommendations should therefore be interpreted as requiring local verification, and identification of the system-level moderators of this variation is a priority for future research.

The limits of these data should be emphasized. TIMSS contains no health outcomes. Victimization and belonging are psychosocial well-being indicators, and no claim is made regarding effects on mental health, service use, or clinical outcomes. Validating multi-informant school safety measurement against mental-health and healthcare-use outcomes—linking these informant-specific indicators to service utilization and to identified need [3,16]—is the necessary next step.

### 4.5. Limitations

The design is cross-sectional; all associations are concurrent and the language of prediction is statistical, not causal. The cross-lagged approach of Benbenishty et al. [13] applied to multi-informant panel data would be the natural next step.

The shared-perspective account above is the most important interpretive limitation and constrains how strongly the central claim can be stated. Rater separation removed shared idiosyncratic variance but not shared school-level construal, and no design available in these data separated the two. A study pairing child self-reports with independently observed incident records would adjudicate this.

Adult and child items differed in referent, content, and level of observation as well as in informant, so that low convergence reflected an unknown combination of these factors. The internal multitrait–multi-informant comparison and the referent-matched single item limited, but did not eliminate, this ambiguity. The teacher item asks about the teacher’s own safety, and readers who regard this as a different construct rather than a different perspective on the same one should interpret the teacher results accordingly; the principal items, which concern student conduct, are less susceptible to this objection and exhibited the same pattern.

Teacher-level reliability remained the weakest element: two independent estimators agreed that a school-level teacher score based on 1.9 raters had a rater-sampling reliability near 0.31, and disattenuation is inadmissible at that level. The principal score had no rater-sampling error but was not error-free; its occasion-specific and role-specific components were not identified here, and the reported corrections should be interpreted accordingly.

Alignment established approximate rather than exact invariance within informants, and the flagging thresholds were conventions. Several blocks were just identified. Alignment provides no information about comparability across informants.

The intervals reported here are model-based cross-system intervals, obtained by resampling education systems and, in sensitivity analyses, schools within them. They are not the design-based intervals TIMSS itself prescribes, which use jackknife replicate weights reproducing the two-stage sampling within each system [46]. The estimand is a cross-system quantity, for which the system is the natural exchangeable unit, and the schools-within-systems bootstrap indicated that within-system sampling variance did not drive these intervals; readers requiring conventional TIMSS design-based standard errors for a single system should compute them using the replicate weights. Between-system heterogeneity in the incongruence slopes ranged from negligible to substantial (I^2^ = 0–62%). The random-effects summary and the dispersion are reported, but the sources of that dispersion were not modeled; system-level moderators constitute a natural extension.

The variance explained by the informant reports was modest in absolute terms—between 1% and 13% of between-school variance depending on the outcome—so the domain gradient describes the division of a limited quantity of explainable variance rather than the proportion of school functioning that these reports capture.

Finally, the extended covariate models lost approximately 14% of schools to principal-questionnaire nonresponse. Because parameters changed minimally between the base and extended specifications, this is unlikely to be consequential; the two specifications are nevertheless reported separately rather than merged.

## 5. Conclusions

The question posed in the title has a two-part answer, corresponding to the two findings this analysis established. First, the characterization of a school as safe depended materially on which informant was surveyed. Adults described schools within a narrow band near the upper limit of the scale whereas children did not, and the two sets of descriptions were close to statistically independent. Neither the concentration of adult reports at the ceiling nor the unreliability of the school-level aggregates accounted for that independence, and it was not attributable to scales lacking comparable meaning across education systems. Informant divergence at the school level is therefore substantive rather than a measurement artifact to be removed by averaging, which is nevertheless the prevailing practice.

Second, and of greater consequence, the divergence was structured. Criterion-related informant validity in school safety measurement was domain-specific: each informant’s report carried greatest weight where the outcome lay within the context that informant observes. Child reports predominated for outcomes describing children’s experience of school; the three informants contributed comparably for externally assessed achievement, where adults’ reports of institutional conditions proved as informative as children’s. Adult reports were therefore neither inaccurate nor redundant, and the domain in which they carried least weight was precisely the domain of greatest relevance to school-health surveillance. The informant reports explain only a modest fraction of between-school variance, so the finding concerns the division of a limited quantity of explainable variance rather than the proportion of school functioning these reports capture; and because the adult and child instruments differ in what they ask about as well as in who answers them, the result bears on the coverage of the adult instruments currently in use as much as on the epistemic position of adult informants. Neither qualification alters the practical implication. The valid surveillance of student well-being, the accurate identification of psychosocial risk, well-targeted prevention, and the equitable allocation of counseling resources all require the child’s own report, because it is precisely the input that adult proxies track least well [3,11,12].

## Figures and Tables

**Table 1:** Sample, informant structure, and measures.

Feature	Value
Education systems	63
Schools	12,955
Students	395,961
Teacher records/unique teachers	27,100/24,887
Schools with all three informants	11,371 (87.8%)
Median students per school (full/split-half)	28/14
Mean unique responding teachers per school	1.92
Random within-school splits averaged	50
Plausible values	5 per domain, carried separately, combined by Rubin’s rule
Uncertainty	percentile bootstrap, 1000 resamples of education systems
Child safety, referent-matched item	ASBG13B: I feel safe at school
Teacher safety (substantive predictor)	ATBG07A, B
Principal safety	ACBG14H, I, J
Child-reported outcomes (split-half B)	victimization; belonging; classroom disorder; absenteeism
Externally assessed outcomes	mathematics and science achievement

*Note.* TIMSS 2023 Grade 4. Within each school, students were randomly divided in two: half A supplied the child-reported predictor and half B the child-reported outcomes, so that no student supplied both the child-reported predictor and a child-reported outcome. Achievement was measured on all sampled students and therefore overlapped with the predictor half; Table S2 reports the same models with achievement computed on half B alone. Teacher records are teacher–class links; aggregates were computed on unique teachers, who were sampled corresponding to teachers associated with the sampled classes rather than a probability sample of school staff.

**Table 2:** Components of reliability, reported separately.

Scale	Internal Consistency (α)	Unique Raters per School	ICC(2)	Direct Split-Half
Child safety (3 items)	0.638	28.0	0.632	0.614
Child safety (single item)	—	27.7	0.580	0.579
Child classroom order	—	28.3	0.795	0.775
Teacher safety (2 items)	0.777	1.92	—	0.314
Teacher–student behavior (3 items)	0.878	1.92	—	0.469
Principal safety problems (3 items)	0.854	1.00	n.a.	n.a.
Principal order problems (3 items)	0.773	1.00	n.a.	n.a.

*Note.* α is the median coefficient alpha across the 63 education systems at the level of the individual respondent. The split-half estimate randomly divided raters within schools and applied the Spearman–Brown formula; teacher entries were computed on unique teachers, and a single teacher’s report correlated 0.202 with that of an independent teacher in the same school. Rater-sampling reliability is undefined for principals, since one principal per school is the population of currently serving principals; this does not make the principal score error-free, and it is observed that an α of 0.854 was used as its residual reliability in all corrections (Table S5). Temporal reliability is not identified in single-occasion data. n.a. = not applicable.

**Table 3:** Response surfaces with teachers and principals treated as separate informants.

*Panel A. Child (Single Item) with Teacher*						
**Outcome**	**b Child [95% CI]**	**b Adult [95% CI]**	**a_3_**	**95% CI**	**95% CI, Schools Resampled Too**	**Adult Coefficient Share [95% CI]**
School belonging	0.378 [0.339, 0.425]	0.046 [0.029, 0.065]	0.332 *	[0.290, 0.377]	[0.283, 0.381]	0.109 [0.071, 0.147]
Peer victimization	−0.222 [−0.273, −0.181]	−0.085 [−0.109, −0.060]	−0.138 *	[−0.192, −0.090]	[−0.199, −0.077]	0.275 [0.207, 0.345]
Classroom disorder	−0.297 [−0.357, −0.251]	−0.087 [−0.113, −0.064]	−0.209 *	[−0.270, −0.154]	[−0.273, −0.145]	0.227 [0.174, 0.291]
Absenteeism	−0.074 [−0.112, −0.042]	−0.075 [−0.099, −0.052]	0.001	[−0.039, 0.034]	[−0.045, 0.047]	0.503 [0.379, 0.634]
Mathematics achievement	0.136 [0.089, 0.183]	0.144 [0.114, 0.174]	−0.008	[−0.062, 0.047]	[−0.071, 0.056]	0.514 [0.413, 0.614]
Science achievement	0.132 [0.083, 0.181]	0.159 [0.132, 0.185]	−0.027	[−0.080, 0.026]	[−0.088, 0.035]	0.546 [0.448, 0.644]
***Panel B. Child (single item) with principal***						
**Outcome**	**b child [95% CI]**	**b adult [95% CI]**	**a_3_**	**95% CI**	**95% CI, schools resampled too**	**Adult coefficient share [95% CI]**
School belonging	0.372 [0.333, 0.421]	0.080 [0.057, 0.107]	0.292 *	[0.241, 0.348]	[0.230, 0.350]	0.177 [0.130, 0.228]
Peer victimization	−0.218 [−0.266, −0.177]	−0.127 [−0.158, −0.099]	−0.091 *	[−0.135, −0.046]	[−0.148, −0.035]	0.368 [0.312, 0.430]
Classroom disorder	−0.292 [−0.350, −0.246]	−0.136 [−0.170, −0.105]	−0.156 *	[−0.212, −0.099]	[−0.218, −0.084]	0.318 [0.259, 0.380]
Absenteeism	−0.074 [−0.112, −0.041]	−0.071 [−0.105, −0.043]	−0.003	[−0.041, 0.041]	[−0.051, 0.049]	0.491 [0.357, 0.643]
Mathematics achievement	0.131 [0.086, 0.176]	0.165 [0.124, 0.206]	−0.035	[−0.089, 0.020]	[−0.097, 0.028]	0.558 [0.463, 0.654]
Science achievement	0.127 [0.079, 0.174]	0.178 [0.137, 0.218]	−0.051	[−0.107, 0.004]	[−0.116, 0.014]	0.584 [0.486, 0.683]
***Panel C. Three-predictor model: division of the explained variance***						
**Outcome**	**LMG share child [95% CI]**	**LMG share teacher [95% CI]**	**LMG share principal [95% CI]**	**R^2^ informants [95% CI]**	**Coefficient share C/T/P**	
School belonging	0.952 [0.916, 0.973]	0.019 [0.010, 0.031]	0.028 [0.012, 0.060]	0.132 [0.109, 0.166]	0.823/0.077/0.100	
Peer victimization	0.746 [0.646, 0.821]	0.114 [0.065, 0.181]	0.140 [0.090, 0.217]	0.055 [0.040, 0.077]	0.579/0.198/0.223	
Classroom disorder	0.807 [0.725, 0.870]	0.093 [0.053, 0.151]	0.100 [0.058, 0.164]	0.082 [0.061, 0.114]	0.628/0.182/0.189	
Absenteeism	0.500 [0.269, 0.700]	0.326 [0.184, 0.497]	0.174 [0.069, 0.348]	0.011 [0.006, 0.019]	0.431/0.339/0.230	
Mathematics achievement	0.436 [0.285, 0.588]	0.286 [0.176, 0.397]	0.278 [0.165, 0.391]	0.045 [0.029, 0.061]	0.392/0.309/0.300	
Science achievement	0.384 [0.234, 0.534]	0.322 [0.214, 0.430]	0.294 [0.177, 0.411]	0.047 [0.031, 0.063]	0.363/0.328/0.308	
*Direct contrasts in the child LMG share*						
Contrast	Difference		95% CI	*p*		
belonging minus mathematics	0.516		[0.376, 0.686]	<0.001		
victimization minus mathematics	0.309		[0.203, 0.425]	<0.001		

*Note.* Point estimates average 50 independent within-school splits. The five plausible values are carried separately through the whole procedure and combined by Rubin’s rule, so achievement intervals include the between-plausible-value component. All other intervals are percentile intervals from 1000 bootstrap resamples of education systems; the adjacent column repeats the interval when schools are also resampled within each drawn system. a_3_ is the slope along the line of incongruence and is reported as a secondary description of directional discrepancy, not as a congruence test; all five polynomial coefficients and all four surface parameters appear in Table S9. The LMG share partitions the variance the three informants jointly explain over and above system fixed effects and the covariate, which are retained in every submodel; the coefficient share compares standardized slope magnitudes and is not a variance partition. Table S8 reports the same decomposition with each informant’s share corrected for its own predictor unreliability. Contrast *p* values are bounded below by 1/(B + 1) = 0.001 with B = 1000 bootstrap replicates. * 95% CI excludes zero.

**Table 4:** Sensitivity of the incongruence slope to weighting and inference scheme.

*Panel A. Child with Teacher*								
Outcome	TIMSS School Weights	Equal System Weights	Unweighted	REML a_3_	95% Hartung–Knapp CI	95% Prediction Interval	τ	I^2^
School belonging	0.322	0.320	0.331	0.326	[0.293, 0.360]	[0.174, 0.479]	0.074	15%
Peer victimization	−0.122	−0.132	−0.137	−0.133	[−0.175, −0.091]	[−0.348, 0.081]	0.105	38%
Classroom disorder	−0.177	−0.202	−0.209	−0.208	[−0.254, −0.163]	[−0.457, 0.040]	0.122	50%
Absenteeism	0.017	0.011	0.001	0.006	[−0.021, 0.033]	[−0.021, 0.033]	0.000	0%
Mathematics achievement	−0.045	−0.019	−0.010	−0.009	[−0.054, 0.036]	[−0.241, 0.223]	0.114	41%
Science achievement	−0.068	−0.037	−0.029	−0.025	[−0.069, 0.018]	[−0.240, 0.190]	0.105	37%
***Panel B. Child with principal***								
**Outcome**	**TIMSS school weights**	**Equal system weights**	**Unweighted**	**REML a_3_**	**95% Hartung–Knapp CI**	**95% prediction interval**	**τ**	**I^2^**
School belonging	0.262	0.286	0.292	0.276	[0.237, 0.315]	[0.086, 0.466]	0.093	25%
Peer victimization	−0.054	−0.084	−0.091	−0.068	[−0.107, −0.029]	[−0.223, 0.086]	0.075	12%
Classroom disorder	−0.121	−0.151	−0.156	−0.118	[−0.162, −0.073]	[−0.346, 0.111]	0.112	35%
Absenteeism	0.050	0.006	−0.003	0.015	[−0.022, 0.052]	[−0.066, 0.096]	0.036	0%
Mathematics achievement	−0.069	−0.040	−0.035	−0.068	[−0.134, −0.003]	[−0.459, 0.322]	0.193	61%
Science achievement	−0.081	−0.053	−0.051	−0.079	[−0.144, −0.014]	[−0.469, 0.311]	0.193	62%

*Note.* All columns average the same 50 splits and, for achievement, the five plausible values. The first three are pooled school-level estimates under different weights, with weighted within-system demeaning. The last five estimate a_3_ separately in each education system with at least 40 schools and combine them by restricted maximum likelihood with Hartung–Knapp confidence intervals and a prediction interval for a new education system [55,56]. τ is the between-system standard deviation. The wide prediction intervals show that a_3_ is expected to vary appreciably across systems, even where the summary estimate is precise.

## Data Availability

The TIMSS 2023 international database analyzed in this study is publicly available from the TIMSS & PIRLS International Study Center at https://timss2023.org (accessed on 1 July 2026) and is archived under https://doi.org/10.58150/IEA_TIMSS_2023_G4_data_edition_1 [62]. No new data were created.

## Supplementary Materials

The following supporting information can be downloaded at https://www.mdpi.com/article/10.3390/healthcare14172848/s1. Table S1: Roster of the 63 education systems; Table S2: Achievement models with achievement computed on split-half B alone; Table S3: Alignment optimization across 63 education systems; Table S4: Measures, response scales, orientation, and aggregation rules; Table S5: Disattenuation Sensitivity to the Principal’s Residual Reliability; Table S6: Errors-in-Variables Sensitivity Under Classical-Error Assumptions; Table S7: Adult Composite as a Secondary Summary: Available-Item Versus Complete-Case; Table S8: Relative-importance decomposition corrected for differential predictor unreliability; Table S9: All polynomial coefficients and surface parameters; Table S10: Monte Carlo error of the bootstrap interval endpoints; Table S11: Coverage of nominal 95% intervals in simulation; Table S12: Latent profile enumeration and incremental validity; Table S13: Alignment of the sixth block, child-reported classroom order; Table S14: Sensitivity to the Measurement Model: Item Means Versus Configural Factor Scores; Table S15: Child–adult convergence under four treatments of the adult ceiling; Table S16: Latent multitrait–multi-informant matrix and model fit; Table S17: System-specific incongruence slopes; Table S18: Shared-rater inflation with rater count held equal; Figure S1: Cross-informant convergence is not a ceiling artifact; Figure S2: Response surfaces with teacher and principal treated separately.

## Abbreviations

The following abbreviations are used in this manuscript:

BIC	Bayesian information criterion
BLRT	Bootstrapped likelihood ratio test
CFI	Comparative fit index
CTC(M−1)	Correlated trait–correlated method minus one
ICC	Intraclass correlation coefficient
IEA	International Association for the Evaluation of Educational Achievement
LMG	Lindeman–Merenda–Gold relative-importance measure
LMR	Lo–Mendell–Rubin
MTMI	Multitrait–multi-informant
REML	Restricted maximum likelihood
RMSEA	Root mean square error of approximation
RSA	Response surface analysis
TIMSS	Trends in International Mathematics and Science Study
TLI	Tucker–Lewis index

## References

[1] Kieling C., Buchweitz C., Caye A., Silvani J., Ameis S.H., Brunoni A.R., Cost K.T., Courtney D.B., Georgiades K., Merikangas K.R. (2024). Worldwide prevalence and disability from mental disorders across childhood and adolescence: Evidence from the Global Burden of Disease Study. JAMA Psychiatry.

[2] Solmi M., Radua J., Olivola M., Croce E., Soardo L., Salazar de Pablo G., Il Shin J., Kirkbride J.B., Jones P., Kim J.H. (2022). Age at onset of mental disorders worldwide: Large-scale meta-analysis of 192 epidemiological studies. Mol. Psychiatry.

[3] Duong M.T., Bruns E.J., Lee K., Cox S., Coifman J., Mayworm A., Lyon A.R. (2021). Rates of mental health service utilization by children and adolescents in schools and other common service settings: A systematic review and meta-analysis. Adm. Policy Ment. Health.

[4] Balasooriya Lekamge R., Jain R., Sheen J., Solanki P., Zhou Y., Romero L., Barry M.M., Chen L., Karim M.N., Ilic D. (2025). Systematic review and meta-analysis of the effectiveness of whole-school interventions promoting mental health and preventing risk behaviours in adolescence. J. Youth Adolesc..

[5] Nakamoto J., Schwartz D. (2010). Is peer victimization associated with academic achievement? A meta-analytic review. Soc. Dev..

[6] Halliday S., Gregory T., Taylor A., Digenis C., Turnbull D. (2021). The impact of bullying victimization in early adolescence on subsequent psychosocial and academic outcomes across the adolescent period: A systematic review. J. Sch. Violence.

[7] Allen K., Kern M.L., Vella-Brodrick D., Hattie J., Waters L. (2018). What schools need to know about fostering school belonging: A meta-analysis. Educ. Psychol. Rev..

[8] Goodenow C. (1993). The psychological sense of school membership among adolescents: Scale development and educational correlates. Psychol. Sch..

[9] Gaffney H., Ttofi M.M., Farrington D.P. (2019). Evaluating the effectiveness of school-bullying prevention programs: An updated meta-analytical review. Aggress. Violent Behav..

[10] Ttofi M.M., Farrington D.P. (2011). Effectiveness of school-based programs to reduce bullying: A systematic and meta-analytic review. J. Exp. Criminol..

[11] Steiner R.J., Sheremenko G., Lesesne C., Dittus P.J., Sieving R.E., Ethier K.A. (2019). Adolescent connectedness and adult health outcomes. Pediatrics.

[12] Wilkins N.J., Krause K.H., Verlenden J.V., Szucs L.E., Ussery E.N., Allen C.T., Stinson J., Michael S.L., Ethier K.A. (2023). School connectedness and risk behaviors and experiences among high school students—Youth Risk Behavior Survey, United States, 2021. MMWR Suppl..

[13] Benbenishty R., Astor R.A., Roziner I., Wrabel S.L. (2016). Testing the causal links between school climate, school violence, and school academic performance: A cross-lagged panel autoregressive model. Educ. Res..

[14] Berkowitz R., Moore H., Astor R.A., Benbenishty R. (2017). A research synthesis of the associations between socioeconomic background, inequality, school climate, and academic achievement. Rev. Educ. Res..

[15] Olweus D. (1993). Bullying at School: What We Know and What We Can Do.

[16] Soneson E., Howarth E., Ford T., Humphrey A., Jones P.B., Thompson Coon J., Rogers M., Anderson J.K. (2020). Feasibility of school-based identification of children and adolescents experiencing, or at-risk of developing, mental health difficulties: A systematic review. Prev. Sci..

[17] Furlong M.J., O’Malley M., Chan M., Dowdy E., Goodwin J.W., Ortiz A., Nylund-Gibson K., Hanson T. (2025). The California Student Wellness Index: Development, validation, and multi-tier applications. Contemp. Sch. Psychol..

[18] Achenbach T.M., McConaughy S.H., Howell C.T. (1987). Child/adolescent behavioral and emotional problems: Implications of cross-informant correlations for situational specificity. Psychol. Bull..

[19] De Los Reyes A., Kazdin A.E. (2005). Informant discrepancies in the assessment of childhood psychopathology: A critical review, theoretical framework, and recommendations for further study. Psychol. Bull..

[20] De Los Reyes A., Augenstein T.M., Wang M., Thomas S.A., Drabick D.A.G., Burgers D.E., Rabinowitz J. (2015). The validity of the multi-informant approach to assessing child and adolescent mental health. Psychol. Bull..

[21] Kraemer H.C., Measelle J.R., Ablow J.C., Essex M.J., Boyce W.T., Kupfer D.J. (2003). A new approach to integrating data from multiple informants in psychiatric assessment and research: Mixing and matching contexts and perspectives. Am. J. Psychiatry.

[22] De Los Reyes A., Thomas S.A., Goodman K.L., Kundey S.M.A. (2013). Principles underlying the use of multiple informants’ reports. Annu. Rev. Clin. Psychol..

[23] Laird R.D., De Los Reyes A. (2013). Testing informant discrepancies as predictors of early adolescent psychopathology: Why difference scores cannot tell you what you want to know and how polynomial regression may. J. Abnorm. Child Psychol..

[24] Mitchell M.M., Bradshaw C.P., Leaf P.J. (2010). Student and teacher perceptions of school climate: A multilevel exploration of patterns of discrepancy. J. Sch. Health.

[25] Konold T., Cornell D., Huang F., Meyer P., Lacey A., Nekvasil E., Heilbrun A., Shukla K. (2014). Multilevel multi-informant structure of the Authoritative School Climate Survey. Sch. Psychol. Q..

[26] Bradshaw C.P., Waasdorp T.E., Debnam K.J., Johnson S.L. (2014). Measuring school climate in high schools: A focus on safety, engagement, and the environment. J. Sch. Health.

[27] Cornell D., Huang F. (2016). Authoritative school climate and high school student risk behavior: A cross-sectional multi-level analysis of student self-reports. J. Youth Adolesc..

[28] Thapa A., Cohen J., Guffey S., Higgins-D’Alessandro A. (2013). A review of school climate research. Rev. Educ. Res..

[29] Wang M.-T., Degol J.L. (2016). School climate: A review of the construct, measurement, and impact on student outcomes. Educ. Psychol. Rev..

[30] Baumsteiger R., Hoffmann J.D., Seibyl J., Rose B., Brackett M.A. (2023). A systematic review of secondary school climate assessments. Educ. Psychol. Rev..

[31] Lüdtke O., Marsh H.W., Robitzsch A., Trautwein U., Asparouhov T., Muthén B. (2008). The multilevel latent covariate model: A new, more reliable approach to group-level effects in contextual studies. Psychol. Methods.

[32] Lüdtke O., Robitzsch A., Trautwein U., Kunter M. (2009). Assessing the impact of learning environments: How to use student ratings of classroom or school characteristics in multilevel modeling. Contemp. Educ. Psychol..

[33] Marsh H.W., Lüdtke O., Nagengast B., Trautwein U., Morin A.J.S., Abduljabbar A.S., Köller O. (2012). Classroom climate and contextual effects: Conceptual and methodological issues in the evaluation of group-level effects. Educ. Psychol..

[34] LeBreton J.M., Senter J.L. (2008). Answers to 20 questions about interrater reliability and interrater agreement. Organ. Res. Methods.

[35] Shrout P.E., Fleiss J.L. (1979). Intraclass correlations: Uses in assessing rater reliability. Psychol. Bull..

[36] Podsakoff P.M., MacKenzie S.B., Lee J.-Y., Podsakoff N.P. (2003). Common method biases in behavioral research: A critical review of the literature and recommended remedies. J. Appl. Psychol..

[37] Podsakoff P.M., MacKenzie S.B., Podsakoff N.P. (2012). Sources of method bias in social science research and recommendations on how to control it. Annu. Rev. Psychol..

[38] Meredith W. (1993). Measurement invariance, factor analysis and factorial invariance. Psychometrika.

[39] Rutkowski L., Svetina D. (2014). Assessing the hypothesis of measurement invariance in the context of large-scale international surveys. Educ. Psychol. Meas..

[40] Vandenberg R.J., Lance C.E. (2000). A review and synthesis of the measurement invariance literature: Suggestions, practices, and recommendations for organizational research. Organ. Res. Methods.

[41] Asparouhov T., Muthén B. (2014). Multiple-group factor analysis alignment. Struct. Equ. Model..

[42] Marsh H.W., Guo J., Parker P.D., Nagengast B., Asparouhov T., Muthén B., Dicke T. (2018). What to do when scalar invariance fails: The extended alignment method for multi-group factor analysis comparison of latent means across many groups. Psychol. Methods.

[43] Muthén B., Asparouhov T. (2018). Recent methods for the study of measurement invariance with many groups. Sociol. Methods Res..

[44] von Davier M., Kennedy A., Reynolds K., Fishbein B., Khorramdel L., Aldrich C., Bookbinder A., Bezirhan U., Yin L. (2024). TIMSS 2023 International Results in Mathematics and Science.

[45] Mullis I.V.S., Martin M.O., von Davier M. (2021). TIMSS 2023 Assessment Frameworks.

[46] von Davier M., Fishbein B., Kennedy A. (2024). TIMSS 2023 Technical Report: Methods and Procedures.

[47] Humberg S., Nestler S., Back M.D. (2019). Response surface analysis in personality and social psychology: Checklist and clarifications for the case of congruence hypotheses. Soc. Psychol. Personal. Sci..

[48] Eid M., Lischetzke T., Nussbeck F.W., Trierweiler L.I. (2003). Separating trait effects from trait-specific method effects in multitrait-multimethod models: A multiple-indicator CT-C(M−1) model. Psychol. Methods.

[49] Eid M., Nussbeck F.W., Geiser C., Cole D.A., Gollwitzer M., Lischetzke T. (2008). Structural equation modeling of multitrait-multimethod data: Different models for different types of methods. Psychol. Methods.

[50] Campbell D.T., Fiske D.W. (1959). Convergent and discriminant validation by the multitrait-multimethod matrix. Psychol. Bull..

[51] Barranti M., Carlson E.N., Côté S. (2017). How to test questions about similarity in personality and social psychology research: Description and empirical demonstration of response surface analysis. Soc. Psychol. Personal. Sci..

[52] Edwards J.R., Parry M.E. (1993). On the use of polynomial regression equations as an alternative to difference scores in organizational research. Acad. Manag. J..

[53] Shanock L.R., Baran B.E., Gentry W.A., Pattison S.C., Heggestad E.D. (2010). Polynomial regression with response surface analysis: A powerful approach for examining moderation and overcoming limitations of difference scores. J. Bus. Psychol..

[54] Grömping U. (2006). Relative importance for linear regression in R: The package relaimpo. J. Stat. Softw..

[55] Hartung J., Knapp G. (2001). On tests of the overall treatment effect in meta-analysis with normally distributed responses. Stat. Med..

[56] IntHout J., Ioannidis J.P.A., Borm G.F. (2014). The Hartung-Knapp-Sidik-Jonkman method for random effects meta-analysis is straightforward and considerably outperforms the standard DerSimonian-Laird method. BMC Med. Res. Methodol..

[57] Wagenmakers E.-J., Wetzels R., Borsboom D., van der Maas H.L.J., Kievit R.A. (2012). An agenda for purely confirmatory research. Perspect. Psychol. Sci..

[58] Masyn K.E., Little T.D. (2013). Latent class analysis and finite mixture modeling. The Oxford Handbook of Quantitative Methods in Psychology: Vol. 2: Statistical Analysis.

[59] Nylund K.L., Asparouhov T., Muthén B.O. (2007). Deciding on the number of classes in latent class analysis and growth mixture modeling: A Monte Carlo simulation study. Struct. Equ. Model..

[60] Bear G.G., Yang C., Pell M., Gaskins C. (2014). Validation of a brief measure of teachers’ perceptions of school climate: Relations to student achievement and suspensions. Learn. Environ. Res..

[61] Koslouski J.B., Chafouleas S.M., Briesch A., Caemmerer J.M., Melo B. (2024). Developing a whole child school screening instrument: Evaluating perceived usability as an initial step in planning for consequential validity. Sch. Ment. Health.

[62] International Association for the Evaluation of Educational Achievement (IEA), TIMSS & PIRLS International Study Center at Boston College (2024). TIMSS 2023 International Database: Trends in International Mathematics and Science Study—Grade 4.

