# Supplementary material for: Whose School Is Safe? Informant-Specific Validity in School Safety and Student Well-Being Assessment Across 63 Education Systems
Source: Healthcare (Basel). 2026 Sep 4;14(17):2848. doi: 10.3390/healthcare14172848 (PMC13565373; doi:10.3390/healthcare14172848)
Supplement: Supplementary file 1 [file healthcare-14-02848-s001.zip › healthcare-4495194-supplementary.pdf]

Supplementary Materials

Whose School Is Safe? Informant-Specific Validity in School Safety and Student Well-Being Assessment Across 63 Education Systems

Georgios Sideridis

This file contains eighteen supplementary tables and two supplementary figures supporting the analyses reported in the main text. All values are produced by the analysis code deposited with the manuscript.

Table S1. Roster of the 63 Education Systems.

Counts are from the analyzed database. “Unique teachers” deduplicates the teacher–class link records on country, school, and teacher identifier. “Nested BM” marks benchmarking entities nested within a participating parent country.

Education system	Schools	Students	Unique teachers	Schools w/ teacher	Schools w/ principal	All three	Nested BM
Abu Dhabi, UAE	286	12558	719	276	235	229	Yes
Albania	108	2374	133	90	92	78	No
Armenia	149	4855	294	145	145	141	No
Australia	283	5470	416	229	239	198	No
Azerbaijan	199	6057	327	178	172	159	No
Bahrain	187	5435	388	187	186	186	No
Belgium (Flemish)	146	4336	290	123	132	112	No
Belgium (French)	165	4875	395	154	149	143	No
Bosnia and Herzegovina	120	3391	236	104	97	89	No
Brazil	796	22130	1145	778	760	744	No
Bulgaria	153	4103	230	152	153	152	No
Canada	659	13716	1011	619	584	563	No
Canada—Quebec	146	3881	257	141	124	122	Yes
Chile	178	4537	274	173	139	138	No
Chinese Taipei	215	5308	499	215	211	210	No
Cyprus	148	4829	388	148	148	148	No

Czech Republic	195	6822	420	195	190	190	No
Côte d’Ivoire	163	4266	295	150	135	130	No
Denmark	158	5166	377	148	137	127	No
Dubai, UAE	201	9294	496	186	173	163	Yes
England	131	4091	203	116	104	96	No
Finland	170	5803	392	169	162	161	No
France	159	4739	337	141	135	129	No
Georgia	156	4411	296	156	154	154	No
Germany	230	4442	353	207	198	185	No
Hong Kong SAR	139	4141	360	131	99	94	No
Hungary	154	5241	345	142	139	133	No
Iran	224	6138	224	222	222	220	No
Ireland	153	4750	239	153	149	149	No
Italy	152	4453	266	150	148	146	No
Japan	141	3875	251	139	140	137	No
Jordan	224	6065	427	224	222	222	No
Kazakhstan	185	6508	327	183	179	177	No
Korea, Rep. of	156	4355	218	144	138	134	No
Kosovo	152	4609	231	137	132	121	No
Kuwait	150	4486	328	149	147	147	No
Latvia	154	4299	326	152	149	147	No
Lithuania	190	4520	250	189	182	182	No
Macao SAR	59	5513	399	59	59	59	No
Montenegro	141	4417	339	138	133	131	No
Morocco	270	7846	306	256	252	248	No
Netherlands	123	3555	188	88	72	61	No
New Zealand	154	4947	404	108	112	87	No
North Macedonia	149	4633	276	114	143	111	No
Norway	160	5301	395	146	136	130	No
Oman	220	8068	377	217	207	204	No
Poland	150	4666	385	149	150	149	No
Portugal	164	5214	323	164	159	159	No

Qatar	279	6412	482	276	269	268	No
Romania	130	3559	195	108	76	71	No
Saudi Arabia	214	5515	426	203	199	190	No
Serbia	158	4349	213	157	157	156	No
Sharjah, UAE	144	6864	387	138	121	117	Yes
Singapore	181	6530	635	181	179	179	No
Slovak Republic	162	4788	328	162	162	161	No
Slovenia	149	4746	269	148	142	142	No
South Africa	285	10424	517	253	233	220	No
Spain	524	10216	785	520	503	499	No
Sweden	160	5139	277	147	138	127	No
Türkiye	141	4541	342	141	139	139	No
United Arab Emirates	755	34842	1933	721	638	617	No
United States	242	9090	517	240	233	231	No
Uzbekistan	166	4457	226	161	164	159	No
**Total (63)**	**12,955**	**395,961**	**24,887**	**12,290**	**11,776**	**11,371**	**4**

Note. The analyzed database contained **four** benchmarking entities nested within a participating parent country: Abu Dhabi, Dubai, and Sharjah within the United Arab Emirates, and Quebec within Canada. Ontario, the fifth TIMSS 2023 benchmarking entity nested within Canada, was not present in this data edition; the sensitivity analysis excluding nested benchmarking entities therefore concerns these four. Kurdistan Region of Iraq is a benchmarking entity, but its parent did not participate, so it is not nested within a participating country.

Table S2. Achievement Models With Achievement Computed on Split-Half B Alone.

Model	Outcome	All sampled students	Split-half B only	b child, all → half B	b adult, all → half B
teacher	Mathematics achievement	−.008	−.015	.136 → .121	.144 → .136
teacher	Science achievement	−.027	−.031	.132 → .118	.159 → .149
principal	Mathematics achievement	−.035	−.042	.131 → .116	.165 → .157
principal	Science achievement	−.051	−.054	.127 → .113	.178 → .167

three-predictor (child LMG share)	Mathematics achievement	.436	.399	—	—
three-predictor (child LMG share)	Science achievement	.384	.358	—	—

Note. Achievement is measured on all sampled students, so for the achievement models the child predictor half is not disjoint from the students contributing the outcome. Re-running with achievement aggregated on split-half B alone makes predictor and outcome rest on disjoint children. For the pairwise models the entries are a_3; for the three-predictor model they are the child LMG share. Coefficients shrink slightly and the child share falls by about four percentage points, so mechanical overlap does not account for the domain gradient.

Table S3. Alignment Optimization Across 63 Education Systems.

Scale	Items	N	Mdn CFI	SD loadings raw → aligned	SD intercepts raw → aligned	% noninvariant	r aligned vs. raw mean
Child safety (student level)	3	355,180	just identified	.076 → .051	.189 → .088	1.9	.986
Teacher safety and security	4	24,383	.860	.110 → .040	.211 → .083	2.0	.982
Teacher student behavior 3	2	4,383 j	ust identified .	105 → .043 .	286 → .057 0	.0 .	999
Principal safety problems	3	11,738	just identified	.238 → .081	.284 → .097	1.9	.989
Principal order problems	3	11,745	just identified	.178 → .082	.300 → .148	5.6	.942
TIMSS teacher scale, 7 items (supplementary)	7	24,383	.793	.104 → .053	.243 → .084	0.8	.996
TIMSS principal scale, 6 items (supplementary)	6	11,705	.882	.206 → .076	.291 → .155	5.3	.934

Note. The sixth informant-by-trait block, child-reported classroom order, is reported separately in Table S13. The teacher safety block here is the four-item safety-and-security block (ATBG07A, B, F, G); the two-item substantive predictor in Table 2 uses only the two items whose referent is safety. Alignment operates **within informant**. Three-item blocks are just identified, so their configural fit is not testable and the alignment result for those blocks describes parameter dispersion

only. Alignment provides no evidence about comparability **across** informants, which is the question the multitrait–multi-informant model addresses.

Table S4. Measures, Response Scales, Orientation, and Aggregation Rules.

All questionnaire items used a four-point scale. Codes 8 (not administered) and 9 (omitted or invalid) were recoded to missing, and any value greater than 4 was set to missing. A respondent-level scale score was the mean of the non-missing constituent items (minimum one valid item). School aggregates of student items were weighted by the total student weight within school; teacher aggregates were unweighted means over unique responding teachers; principal scores were single-respondent values; achievement school means were weighted by the total student weight and computed on all sampled students.

Construct (informant)	Items	Response scale	Orientation after recoding	Score range
Safety (child)	ASBG13B, D, G: *I feel safe at school*; *my teachers care about me*; *students at my school like each other*	1 = agree a lot … 4 = disagree a lot	Reversed; higher = safer	1–4
Safety, referent-matched item (child)	ASBG13B: *I feel safe at school*	1 = agree a lot … 4 = disagree a lot	Reversed; higher = safer	1–4
Order (child)	ASBM04E, F; ASBS09E, F: teacher must keep telling students to follow rules; other students disrupt the lesson (mathematics and science)	1 = every/almost every lesson … 4 = never	Higher = more orderly	1–4
Safety and security (teacher)	ATBG07A, B (predictor); F, G added for the alignment block	1 = agree a lot … 4 = disagree a lot	Reversed; higher = safer	1–4
Student behavior (teacher)	ATBG07C, D, E: students behave well; are respectful; respect property	1 = agree a lot … 4 = disagree a lot	Reversed; higher = more orderly	1–4
Safety problems	ACBG14H, I, J: intimidation among	1 = not a problem … 4 =	Reversed; higher =	1–4

(principal)	students; physical fights; intimidation of teachers	serious problem	fewer problems	
Order problems (principal)	ACBG14A, B, C: arriving late; absenteeism; classroom disturbance	1 = not a problem … 4 = serious problem	Reversed; higher = fewer problems	1–4
Peer victimization (child, outcome)	ASBG14A–K (11 items)	1 = at least once a week … 4 = never	Reversed; higher = more victimization	1–4
School belonging (child, outcome)	ASBG13A, C, E, F (disjoint from the safety predictor)	1 = agree a lot … 4 = disagree a lot	Reversed; higher = more belonging	1–4
Classroom disorder (child, outcome)	Reverse of the child order items	1–4	Higher = more disorder	1–4
Absenteeism (child, outcome)	ASBG08: how often absent from school	ordinal frequency	Higher = more frequent absence	scale points
Mathematics achievement (external)	Five plausible values (ASMMAT01–05)	IEA IRT scale	Higher = higher achievement	≈ continuous
Science achievement (external)	Five plausible values (ASSSCI01–05)	IEA IRT scale	Higher = higher achievement	≈ continuous

Note. Safety and order predictors and all outcomes were standardized within education system before analysis. Consistent with this coding, model slopes were negative for the adverse outcomes (peer victimization, classroom disorder, absenteeism) and positive for belonging and achievement.

Table S5. Disattenuation Sensitivity to the Principal's Residual Reliability.

Scenario	Principal reliability	Mean convergent r	Mean same-informant r	Ratio	Inadmissible entries	Child–principal safety	Principal within-informant
Observed internal consistency	0.854	.286	.856	3.00	1	.181	.980

(primary)							
Upper bound: no residual error	1.000	.268	.764	2.85	1	.167	.796
Lower bound .80	0.800	.286	.864	3.02	1	.187	.995
Lower bound .70	0.700	.298	.732	2.46	2	.200	1.137

Note. Uncorrected values are .179, .636 and a ratio of 3.56. The principal school score has no rater-sampling error, but that is one variance component, not a total reliability. The primary scenario therefore assigns the principal its observed internal consistency (α = .854 for safety, .773 for order); 1.00 is reported only as an explicit upper bound, and .80 and .70 as lower bounds representing unmodeled occasion and role error. Child and teacher aggregates keep their directly estimated rater-sampling reliabilities. Corrections are computed under classical-error assumptions and are sensitivity analyses, not point estimates; entries exceeding unity are counted rather than truncated.

Table S6. Errors-in-Variables Sensitivity Under Classical-Error Assumptions.

Child predictor	Outcome	b child (obs.)	b adult (obs.)	b child (corr.)	b adult (corr.)	Ratio (obs.)	Ratio (corr.)
3-item	Peer victimization	−.278	−.100	−.632	−.086	2.77	7.38
3-item	School belonging	.412	.058	.946	−.001	7.09	—
3-item	Classroom disorder	−.304	−.099	−.692	−.078	3.08	8.87
3-item	Absenteeism	−.088	−.071	−.196	−.083	1.23	2.35
3-item	Mathematics achievement	.194	.142	.434	.161	1.37	2.70
3-item	Science achievement	.206	.153	.461	.174	1.35	2.65
1-item	Peer victimization	−.225	−.107	−.554	−.101	2.11	5.51
1-item	School belonging	.372	.064	.927	.007	5.81	—
1-item	Classroom disorder	−.268	−.104	−.662	−.086	2.59	7.68
1-item	Absenteeism	−.061	−.075	−.144	−.092	0.81	1.57
1-item	Mathematics	.145	.147	.348	.175	0.98	1.99

	achievement						
1-item	Science achievement	.145	.159	.346	.192	0.91	1.80

Note. Correction subtracts estimated error variance from the diagonal of the predictor covariance matrix. The adult composite is used here. Reliabilities: child half-sample predictor .443 (3 items) and .408 (single item); adult composite .716 under the upper-bound assumption of an error-free principal score, which Table S5 shows to be optimistic. These results are a sensitivity analysis under classical-error assumptions and should not be read as point estimates.

Table S7. Adult Composite as a Secondary Summary: Available-Item Versus Complete-Case.

Outcome	Composite rule	N	b child	b adult	a_3	Adult share
Peer victimization	(available-item)	12,576	−.230	−.118	−.112	.339
School belonging	(available-item)	12,573	.387	.069	.318	.152
Classroom disorder	(available-item)	12,388	−.297	−.123	−.175	.292
Absenteeism	(available-item)	12,578	−.064	−.071	.007	.526
Mathematics achievement	(available-item)	12,606	.137	.182	−.044	.569
Science achievement	(available-item)	12,606	.133	.199	−.066	.599
Peer victimization	(complete-case)	11,342	−.224	−.118	−.106	.345
School belonging	(complete-case)	11,341	.384	.070	.313	.155
Classroom disorder	(complete-case)	11,157	−.291	−.126	−.164	.303
Absenteeism	(complete-case)	11,343	−.067	−.071	.004	.515
Mathematics achievement	(complete-case)	11,367	.128	.180	−.052	.584
Science achievement	(complete-case)	11,367	.124	.199	−.075	.616

Note. The available-item composite averages whichever adult reports are present, which is why its N exceeds the principal-report N; 6.8% of schools contributed a

teacher report only and 2.9% a principal report only. The complete-case composite is restricted to the 88.0% of schools with both adult reports. The composite is reported only as a secondary summary; Table 3 carries the primary analyses.

Table S8. Relative-Importance Decomposition Corrected for Differential Predictor Unreliability.

Outcome	Informant	Observed share	Corrected (α = .854)	ρ = .70	ρ = 1.00
School belonging	Child	95	94	94	94
	Teacher	2	4	4	4
	Principal	3	2	3	2
Peer victimization	Child	75	71	69	71
	Teacher	11	20	20	20
	Principal	14	9	11	8
Classroom disorder	Child	81	77	76	77
	Teacher	9	17	16	17
	Principal	10	7	8	6
Absenteeism	Child	50	40	39	40
	Teacher	33	51	50	52
	Principal	17	10	11	8
Mathematics achievement	Child	44	37	35	37
	Teacher	29	47	45	48
	Principal	28	17	20	15
Science achievement	Child	38	31	30	32
	Teacher	32	51	50	53
	Principal	29	17	20	15

Note. Shares are percentages of the variance the three informant scores jointly explain over and above education-system fixed effects and the covariate; they are the LMG shares of Table 3, Panel C. Correction divides each informant's share by that informant's predictor reliability and renormalizes, which under the near-orthogonality of the informant scores (median pairwise r = .078 to .116) closely approximates a full errors-in-variables refit. Reliabilities are .61 for the child aggregate and .314 for the teacher aggregate (Table 2); ρ denotes the principal's assumed residual reliability, taken at its observed internal consistency of .854 and at the .70 and 1.00 bounds. No corrected share moves by more than three percentage points across those bounds. Because each informant's predictor is the same variable in all six outcome models, its reliability is constant across outcomes, so predictor unreliability shifts the level of a share but cannot generate a difference between outcomes. Columns may not sum to 100 because of rounding. The correction is

reported as a bound rather than as an estimate: classical disattenuation is untenable at a reliability of .314.

Table S9. All Polynomial Coefficients and Surface Parameters.

Adult informant	Outcome	b_1	b_2	b_3	b_4	b_5	a_1	a_2	a_3	a_4
teacher	School belonging	.378	.046	.029	−.000	.005	.424	.034	.332	.034
teacher	Peer victimization	−.222	−.085	−.023	−.021	−.006	−.307	−.050	−.138	−.008
teacher	Classroom disorder	−.297	−.087	−.052	−.015	−.008	−.385	−.075	−.209	−.045
teacher	Absenteeism	−.074	−.075	−.001	−.010	−.007	−.150	−.018	.001	.002
teacher	Mathematics achievement	.136	.144	−.011	.028	.016	.280	.034	−.008	−.023
teacher	Science achievement	.132	.159	−.008	.031	.020	.290	.043	−.027	−.019
principal	School belonging	.372	.080	.027	.000	.021	.453	.048	.292	.048
principal	Peer victimization	−.218	−.127	−.024	−.005	−.027	−.345	−.056	−.091	−.046
principal	Classroom disorder	−.292	−.136	−.050	−.001	−.032	−.428	−.082	−.156	−.081
principal	Absenteeism	−.074	−.071	−.003	.000	−.015	−.145	−.018	−.003	−.018
principal	Mathematics achievement	.131	.165	−.011	.003	.030	.296	.022	−.035	.016
principal	Science achievement	.127	.178	−.008	−.000	.033	.304	.025	−.051	.025

Note. b_1 and b_2 are the linear child and adult terms, b_3 and b_5 the quadratics and b_4 the interaction. $a_1 = b_1 + b_2$; $a_2 = b_3 + b_4 + b_5$; $a_3 = b_1 - b_2$; $a_4 = b_3 - b_4 + b_5$. Estimates average 50 splits and, for achievement, five plausible values. The surface is reported as a secondary description of directional discrepancy; congruence is not inferred from any subset of these parameters.

Table S10. Monte Carlo Error of the Bootstrap Interval Endpoints.

Outcome	Lower limit	MCSE	Upper limit	MCSE
School belonging	.916	.002	.973	.001
Peer victimization	.646	.009	.821	.003
Classroom disorder	.725	.004	.870	.003
Absenteeism	.269	.008	.700	.010

Mathematics achievement	.285	.010	.588	.006
Science achievement	.234	.005	.534	.006

Note. Child LMG share, 1,000 bootstrap resamples of education systems. The Monte Carlo standard errors are obtained by resampling the bootstrap replicates themselves 400 times and taking the standard deviation of the resulting percentiles; they describe how much of the reported interval is resampling noise rather than sampling uncertainty. All are an order of magnitude smaller than the intervals themselves, so the reported endpoints are stable at the precision at which they are reported. The lower and upper limits shown are the reported Table 3 endpoints; for the two achievement outcomes these are the Rubin-combined endpoints (bootstrap plus plausible-value uncertainty), which is why they exceed the bootstrap-only endpoints by up to the reported Monte Carlo standard error.

Table S11. Coverage of Nominal 95% Intervals in Simulation.

Interval procedure	Coverage	Mean width
A. Rubin's rules over splits (splits treated as multiple	-imputation	completions) 1.000 .151
B. Single split, cluster-robust standard errors	.933	.127
C. Split average with system bootstrap (adopted here)	.960	.108

Note. 225 replications. A finite population of 300 education systems was generated once with schools nested in systems and students within schools; the estimand (.101) is the value the split-averaged estimator takes in that population. Each replication draws 30 systems. Treating random splits as multiple-imputation completions is markedly conservative: it never missed the estimand and its intervals are about 39% wider than necessary. A single split slightly under-covers. The adopted procedure is close to nominal and the narrowest of the three.

Table S12. Latent Profile Enumeration and Incremental Validity.

k	BIC	Entropy	Smallest class	LMR LR	LMR p	BLRT p
1	95,956	—	100.0%	—	—	—
2	87,131	.697	48.7%	8,890.5	< .001	.02
3	85,305	.625	23.3%	1,891.9	< .001	.02
4	83,927	.598	15.2%	1,443.1	< .001	.02
5	83,436	.601	6.8%	—	—	—
6	82,188	.604	9.3%	—	—	—
7	80,296	.626	5.7%	—	—	—

Outcome	ΔR^2 (k = 3)	ΔR^2 (k = 4)	ΔR^2 (k = 5)
Mathematics achievement	.0000	.0005	.0011
Science achievement	.0001	.0006	.0014
Absenteeism	.0004	.0003	.0004
School belonging	.0001	.0001	.0001
Classroom disorder	.0002	.0007	.0006
Peer victimization	.0001	.0005	.0002

Note. N = 11,371 schools. BIC declines monotonically with no elbow, entropy peaks at k = 2 and falls thereafter, and both likelihood ratio tests reject the k − 1 model at every step computed, with no stopping point — the pattern expected when a continuous distribution is sliced rather than a class structure recovered. Maximum incremental R^2 over the continuous informant scores across all outcomes and solutions is 0.0014.

Table S13. Alignment of the Sixth Block, Child-Reported Classroom Order.

Quantity	Value
Systems	62
Items	4
N	330,240
Median CFI	.876
Median RMSEA	.319
SD loadings raw → aligned	.091 → .074
SD intercepts raw → aligned	.208 → .067
% noninvariant	1.2
r aligned vs. raw mean	.993

Note. This block completes the six informant-by-trait blocks of the multitrait–multi-informant model. The one-factor configural fit is poor on the RMSEA (median RMSEA = .319) because the four items span mathematics and science lessons and the block is short, which inflates the RMSEA at low degrees of freedom; parameter dispersion after alignment is nonetheless comparable to the other blocks, so the block's alignment result is read narrowly.

Table S14. Sensitivity to the Measurement Model: Item Means Versus Configural Factor Scores.

Outcome	Measure	N	b child	b adult	a_1	a_3	a_4

Peer victimization	item means	12,580	−.273	−.111	−.384	−.163	−.010
Peer victimization	factor scores	12,574	−.272	−.111	−.383	−.160	−.006
School belonging	item means	12,577	.418	.063	.481	.355	.026
School belonging	factor scores	12,571	.417	.068	.486	.349	.024
Mathematics achievement	item means	12,610	.181	.174	.355	.007	−.036
Mathematics achievement	factor scores	12,604	.182	.149	.331	.033	−.044
Science achievement	item means	12,610	.188	.189	.377	−.002	−.027
Science achievement	factor scores	12,604	.188	.164	.352	.025	−.034

Note. School-level agreement between item means and configural factor scores was r = .983 (child), .996 (principal) and .791 (teacher; the factor-score version uses four items where the mean uses two). Adult composite.

Table S15. Child–Adult Convergence Under Four Treatments of the Adult Ceiling.

Informant pair	Share at ceiling	Pearson r	Non-ceiling schools	Censored-normal ML	Spearman	Systems with censored r < .20
Child with teacher	.475	.078	.042	.064	.081	60 of 63
Child with principal	.405	.092	.030	.081	.123	56 of 63

Note. Medians across the 63 systems. The censored-normal estimator models the adult report as a normally distributed latent variable right-censored at the scale maximum.

Table S16. Latent Multitrait–Multi-Informant Matrix and Model Fit.

Factor	1	2	3	4	5	6
1. Child safety	—	.505	.120	.246	.131	.145
2. Child order	.505	—	.139	.288	.141	.176
3. Teacher safety	.120	.139	—	.608	.131	.122
4. Teacher order	.246	.288	.608	—	.195	.227
5. Principal safety	.131	.141	.131	.195	—	.796
6. Principal order	.145	.176	.122	.227	.796	—

Model	χ^2	df	CFI	TLI	RMSEA
M1 single factor	70,208.0	135	.381	.298	.216
M2 two traits	63,629.5	134	.439	.359	.207
M3 three informants	18,204.9	132	.840	.815	.111
M4 CTC(M-1)	11,128.0	122	.903	.878	.090
M5 Six informant × trait factors	5,590.6	120	.952	.938	.064

Note. N = 11,084 schools. Mean same-trait, different-informant r = .179; mean different-trait, same-informant r = .636.

Table S17. System-Specific Incongruence Slopes.

Child predictor	Outcome	Systems	Median a_3	% child-dominant	IQR
3-item	Peer victimization	62	−.139	85.5%	[-0.260, -0.061]
3-item	School belonging	62	.301	100.0%	[0.241, 0.447]
3-item	Classroom disorder	61	−.225	85.2%	[-0.349, -0.096]
3-item	Absenteeism	62	.001	48.4%	[-0.136, 0.121]
3-item	Mathematics achievement	62	−.018	51.6%	[-0.157, 0.111]
3-item	Science achievement	62	−.013	51.6%	[-0.155, 0.109]
1-item	Peer victimization	62	−.106	75.8%	[-0.224, -0.002]
1-item	School belonging	62	.287	100.0%	[0.204, 0.384]
1-item	Classroom disorder	61	−.180	78.7%	[-0.328, -0.006]
1-item	Absenteeism	62	.003	50.0%	[-0.093, 0.117]
1-item	Mathematics achievement	62	−.049	62.9%	[-0.186, 0.068]
1-item	Science achievement	62	−.042	62.9%	[-0.209, 0.072]

Note. Systems with at least 80 schools; adult composite. '% child-dominant' is the share of systems in which the incongruence slope runs in the direction of child-report dominance.

Table S18. Shared-Rater Inflation With Rater Count Held Equal.

Outcome	Child predictor	Shared raters	Independent raters	Inflation
Peer victimization	single item	−.282 [−.301, −.267]	−.198 [−.213, −.176]	43% [29%, 63%]
School belonging	single item	.686 [.678, .697]	.355 [.338, .376]	93% [82%, 104%]
Classroom disorder	single item	−.314 [−.331, −.297]	−.261 [−.279, −.245]	20% [10%, 33%]
Absenteeism	single item	−.100 [−.117, −.086]	−.064 [−.081, −.050]	56% [19%, 126%]
Peer victimization	3 items	−.354 [−.371, −.340]	−.240 [−.257, −.223]	48% [35%, 59%]
School belonging	3 items	.774 [.765, .783]	.388 [.364, .407]	100% [90%, 113%]
Classroom disorder	3 items	−.338 [−.355, −.324]	−.293 [−.308, −.275]	15% [7%, 27%]
Absenteeism	3 items	−.144 [−.162, −.124]	−.096 [−.113, −.075]	51% [18%, 96%]

Note. Both predictors are half-sample aggregates, so the two conditions differ only in whether predictor and outcome come from the same children. Each cell averages the two directions (A→A and B→B for shared raters; A→B and B→A for independent raters) and is summarized over 200 random within-school splits; brackets give the 2.5th and 97.5th percentiles across splits.

Figure S1. Cross-Informant Convergence Is Not a Ceiling Artifact.

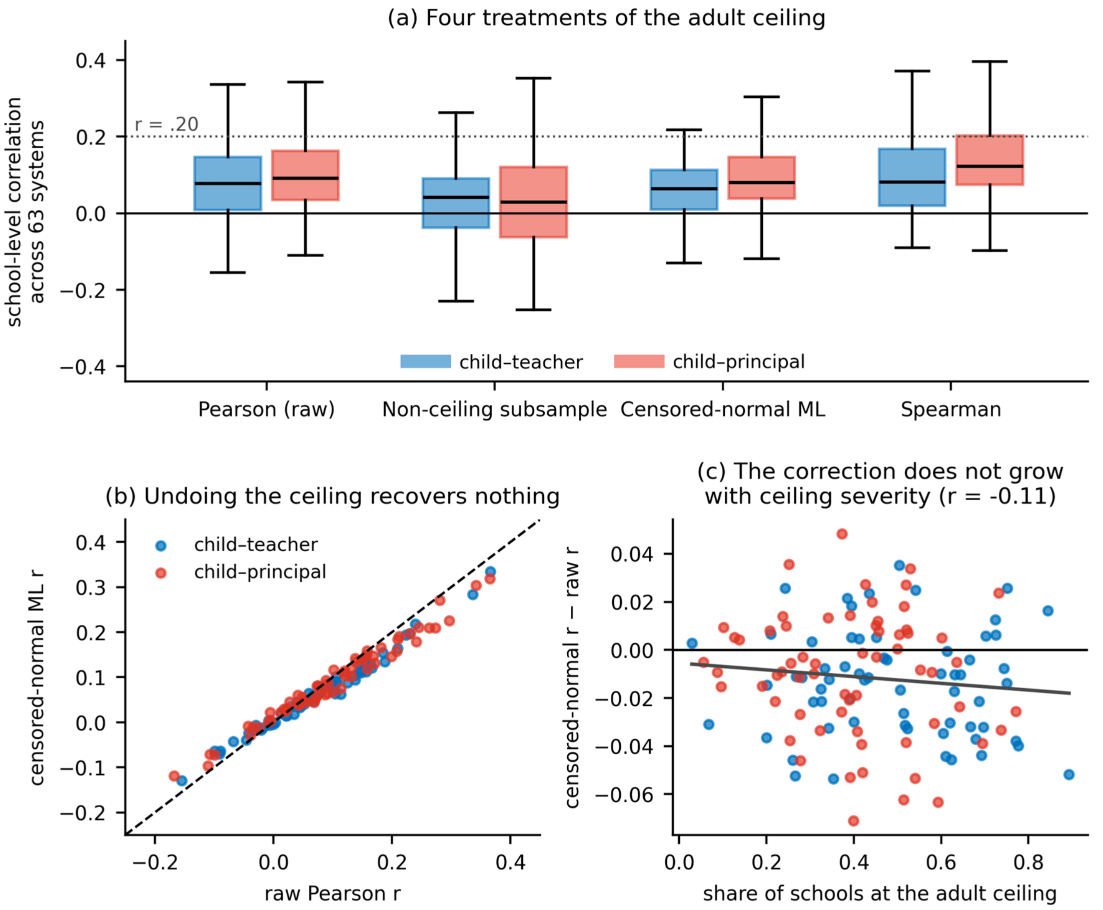

Note. (a) Distribution across 63 education systems of the school-level child–adult safety correlation under four estimators. (b) Censored-normal estimate against the raw Pearson correlation, with the identity line. (c) Size of the censored-normal correction against ceiling severity; if truncation were attenuating the correlations the correction would grow with severity.

Figure S2. Response Surfaces With Teacher and Principal Treated Separately.

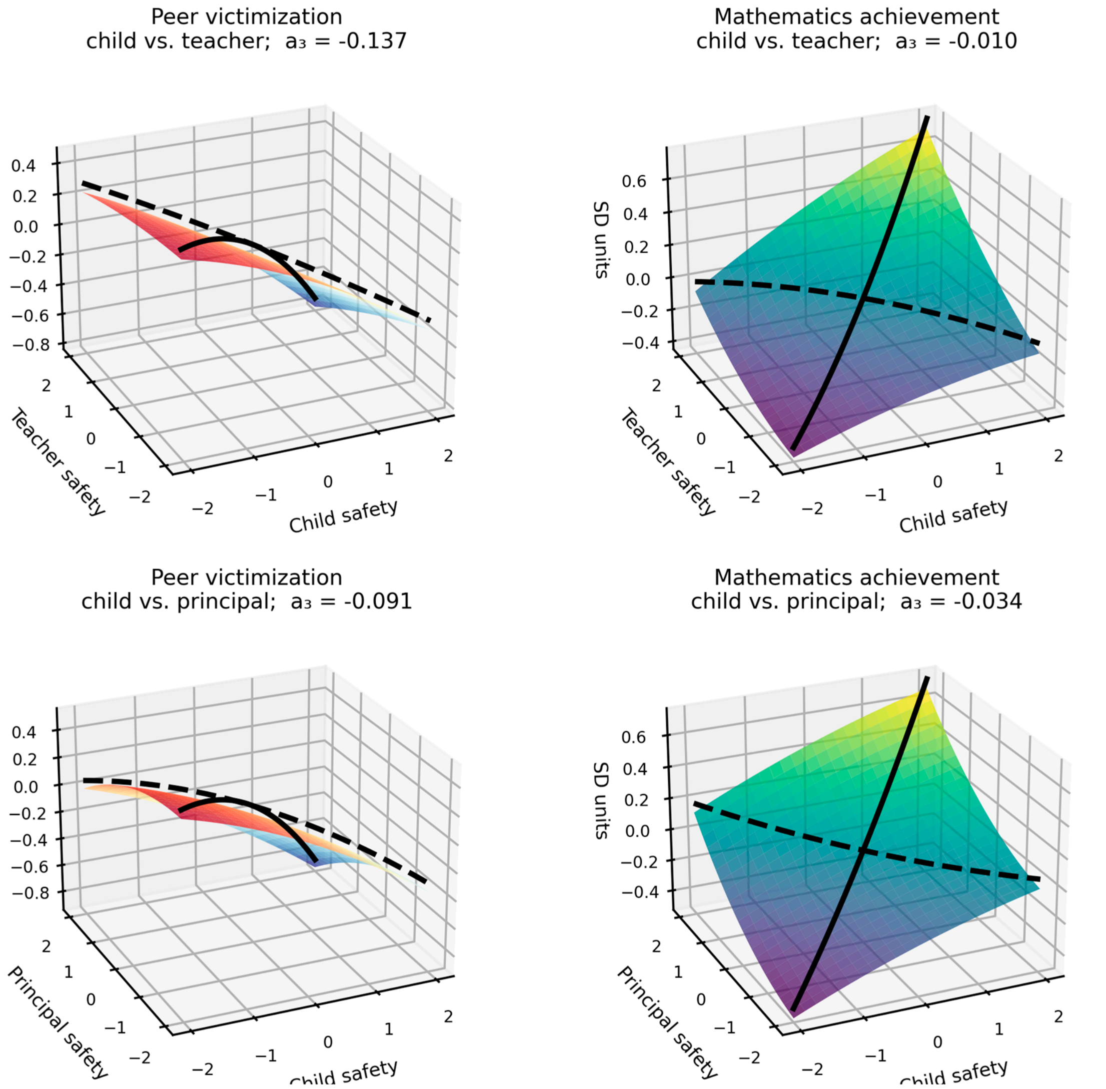

Note. Solid line = line of congruence; dashed = line of incongruence. Child predictor is the referent-matched single item. Surface parameters plotted here use the unweighted pooled specification (Table 4); they differ from the weighted Table 3 / Table S9 estimates by at most .002 (e.g., teacher peer-victimization a_3 = −.137 here vs. −.138 in Table S9). For peer victimization the surface tilts along the child axis against both adult informants; for mathematics achievement it is close to a symmetric plane.
